# Spectrum Degradation of Hippocampal LFP During Euthanasia

**DOI:** 10.3389/fnsys.2021.647011

**Published:** 2021-04-23

**Authors:** Yuchen Zhou, Alex Sheremet, Jack P. Kennedy, Nicholas M. DiCola, Carolina B. Maciel, Sara N. Burke, Andrew P. Maurer

**Affiliations:** ^1^Engineering School of Sustainable Infrastructure and Environment, University of Florida, Gainesville, FL, United States; ^2^Department of Neuroscience, McKnight Brain Institute, University of Florida, Gainesville, FL, United States; ^3^Division of Neurocritical Care, Department of Neurology, University of Florida, Gainesville, FL, United States; ^4^Department of Biomedical Engineering, University of Florida, Gainesville, FL, United States

**Keywords:** local field potential, hippocampus, euthanasia, spectrum evolution, power law spectrum

## Abstract

The hippocampal local field potential (LFP) exhibits a strong correlation with behavior. During rest, the theta rhythm is not prominent, but during active behavior, there are strong rhythms in the theta, theta harmonics, and gamma ranges. With increasing running velocity, theta, theta harmonics and gamma increase in power and in cross-frequency coupling, suggesting that neural entrainment is a direct consequence of the total excitatory input. While it is common to study the parametric range between the LFP and its complementing power spectra between deep rest and epochs of high running velocity, it is also possible to explore how the spectra degrades as the energy is completely quenched from the system. Specifically, it is unknown whether the 1/f slope is preserved as synaptic activity becomes diminished, as low frequencies are generated by large pools of neurons while higher frequencies comprise the activity of more local neuronal populations. To test this hypothesis, we examined rat LFPs recorded from the hippocampus and entorhinal cortex during barbiturate overdose euthanasia. Within the hippocampus, the initial stage entailed a quasi-stationary LFP state with a power-law feature in the power spectral density. In the second stage, there was a successive erosion of power from high- to low-frequencies in the second stage that continued until the only dominant remaining power was <20 Hz. This stage was followed by a rapid collapse of power spectrum toward the absolute electrothermal noise background. As the collapse of activity occurred later in hippocampus compared with medial entorhinal cortex, it suggests that the ability of a neural network to maintain the 1/f slope with decreasing energy is a function of general connectivity. Broadly, these data support the energy cascade theory where there is a cascade of energy from large cortical populations into smaller loops, such as those that supports the higher frequency gamma rhythm. As energy is pulled from the system, neural entrainment at gamma frequency (and higher) decline first. The larger loops, comprising a larger population, are fault-tolerant to a point capable of maintaining their activity before a final collapse.

## 1. Introduction

For over five decades, it has been evident that hippocampal local-field potential (LFP) activity is strongly correlated with behavior (Vanderwolf, 1969; Buzsaki, 2005). The most prominent feature of hippocampal LFP, the 8–9 Hz theta rhythm, is reported to increase in power with increasing running speed (Whishaw and Vanderwolf, 1973; Morris and Hagan, 1983) and change its shape from sinusoidal to sawtooth waves (Green and Petsche, 1961; Stumpf, 1965; Buzsaki et al., 1983; Terrazas et al., 2005) associated with the development of high order theta harmonics during faster movement (Leung, 1982; Leung et al., 1982; Sheremet et al., 2016; Zhou et al., 2019). Apart from theta, the power of the higher frequency gamma rhythm (60–120 Hz) also increases with respect to running speed (Chorbak and Buzsaki, 1998; Chen et al., 2011; Ahmed and Mehta, 2012; Kemere et al., 2013). The increase in gamma power at faster running speeds is also associated with enhanced theta-gamma coupling (Sheremet et al., 2018). These studies imply that when the rat is in an active behaving state, the hippocampus receives strong barrages of synaptic input (Shu et al., 2003), giving rise to an organization of activity across all spatial and temporal scales (Sheremet et al., 2020).

The hippocampal LFP is often decomposed through Fourier analysis, providing the power spectral density. The power spectra density takes the form of Amplitude = 1/frequency^α, where alpha falls is a value between zero and two (characteristic of a pink noise spectra; Buzsaki, 2006). While this decomposition treats different frequency bands as independent signals, the theta and gamma oscillations are not isolated rhythms. In addition to being coupled to each other, theta and gamma also exist against a “background” of activity. The nature of this background is not well-understood, it is often attributed to either the result of interacting oscillators (Buzsaki, 2006) or the consequence of a broadband, arrhythmic activity that is distinct from oscillations (Hesse and Thilo, 2014). Importantly, similar to theta and gamma, the slope of the spectra that is attributed to the background has also been found to change with increasing running speed (Sheremet et al., 2019b), suggesting that it is sensitive to increased synaptic currents and may be interrelated with the rhythms that are often ascribed as being independent from the background spectra.

One explanation for why power is lower at higher frequencies is the energy cascade across multiple oscillators. Precisely, axonal conduction delay and synaptic time constants determine the frequency in which the populations of neurons can rhythmically engage. Faster oscillations require rapid communication, suggesting that a rhythm like gamma would be local, generated by a small population of neurons. The organization of a larger pool of neurons is rate-limited by communication. Global oscillations, like the theta rhythm which engages a larger pool of neurons, will take the form of reciprocal volleys or traveling waves at a slower frequency. As theta engages a larger neuronal population relative to the mechanisms that support gamma, the slow rhythm is approximately one order of magnitude larger than gamma (Buzsaki and Draguhn, 2004). Importantly, these oscillators—among many others- are not independent but one and the same as synapses do not segregate transmembrane currents to specific frequencies but contribute to many bands simultaneously (Bullock et al., 1995; Sheremet et al., 2019a). The intertwining of the rhythmic mechanism, with the large loops (low frequency) providing then energy for the smaller loops (high frequency), gives rise to spatio-temporal interactions that manifest as a 1/f “pink” power spectral density (Buzsaki, 2006). This “energy cascade” hypothesis has roots in self-organized criticality (Bak et al., 1988) and was formalized by Buzsaki concerning mammalian LFP. Within the last few years, the energy cascade hypothesis has been recovered in terms of the classical physics description of turbulence (Sheremet et al., 2019b; Deco and Kringelbach, 2020) which has been cleverly wrangled into a simple verse: *Big whorls have little whorls Which feed on their velocity, And little whorls have lesser whorls And so on to viscosity* (Richardson, 2007). However, other theories regarding the organization of the power spectra suggest that there are two independent biological processes that support aperiodic noise (the 1/f slope) and the rhythmic frequencies, the “peaks” above the slope (He, 2014; Donoghue et al., 2020). These studies consider the log-log linear components of the power spectra where no clear peak above the slope is evident as being carried by either the occurrence of spikes relative to non-preferred LFP oscillation phases (Tort et al., 2010; Lepage et al., 2011) or the desynchronization of spikes within a population of cells (Pozzorini et al., 2013; Voytek and Knight, 2015; Voytek et al., 2015). In sorting out these theories, it should be considered that perhaps the field has placed too much emphasis on the literal interpretation of the power spectral density (calculated by Fourier, Wavelet, or otherwise), which ascribes a single power-to-frequency value suggesting an independence between sinusoidal oscillations of different frequencies. Rather, we should be reminded that the LFP represents the propagation and distribution of activity into densely recurrent networks of neurons, generating the spatial-temporal dynamics responsible for the organization of behavior (Hebb, 1949; Northoff et al., 2019; Atasoy et al., 2020; Nadel and Maurer, 2020). As the nervous system does not work in the currency of pure rhythms vs. noise, but rather dynamics spatio-temporal patterns, this challenges the degree to which we can interpret and make divisions of neurobiological processes based on parameterizing power spectral densities.

Nevertheless, both visual observations and power spectral densities reveal that low-frequency rhythms are larger in amplitude than higher frequency oscillations. Moreover, the 1/f slope of the spectra is a consistent feature of the LFP with the 1/f slope present during sleep and quiescent periods (for instance, see Figure 4 of Buzsáki et al., 2003). The persistence of the 1/f slope across all behavioral states suggests that the nervous system is constantly propagating activity, with large populations of reentrantly connected populations perpetually cascading activity into smaller recurrent networks. Thus, manipulating the spectra require approaches that alter a large swath of synaptic inputs, such as localized cooling (Petersen and Buzsáki, 2020), focal brain lesion (Mitchell et al., 1982; Buzsaki et al., 1987; Bragin et al., 1995; Fyhn et al., 2004), and study the dying brain. In the current study, we investigated the spectra against the background of euthanasia by barbiturate overdose, in which the spectra degrades to a complete collapse.

Prior studies on LFP changes with euthanasia have observed a surge of global and highly coherent gamma oscillation after cardiac arrest (Borjigin et al., 2013). This has led to studies exploring hippocampal physiology in the context of near-death experience (Parnia and Fenwick, 2002), theoretically supported by bursts of high-frequency activity (Zhang et al., 2019). While near-death experience is interesting, this topic is beyond the scope of the present study. Rather, we explored the changes from the perspective of the turbulence hypothesis (Sheremet et al., 2019b; Deco and Kringelbach, 2020). If slow frequency perturbations provide the energy to drive higher frequency oscillations, then we predicted that when the system is significantly challenged, the high-frequency rhythms and the 1/f slope will be the first to be compromised. Low frequency activities, being generated by larger more distributed populations, will be more robust, maintaining power for an extended period of time. In accord with this, we observed a steepening of the 1/f carried by a recession of high-frequency power. At end stage, the LFP was mainly characterized by low frequency, high amplitude activity prior to the final collapse of all activity. This phenomenon was observed across the hippocampus and entorhinal cortex, although the spectral integrity persisted in the hippocampus for relatively longer.

## 2. Materials and Methods

### 2.1. Subjects and Behavioral Training

All behavioral procedures were performed following protocols approved by the Institutional Animal Care and Use Committee at the University of Florida as well as those set forth by the National Institute of Health. The present study consisted of five male hybrid Fisher344-Brown Norway rats (Taconic) ranging from 4 to 10 months of age (r730, r782, r829, r889, and r1074). Animals were singly housed and allowed to acclimate for 1 week after arrival. The colony room maintained a reversed 12–12 h light-dark cycle with all behavior taking place during the dark period. Behavioral shaping began with training animals to run counterclockwise on a circular track one meter in diameter for a food reward (pieces of cereal marshmallow, Medley Hills Farm, Ohio). During this time, the animal's weights were slowly reduced to 85% of their ad lib. weight. Once a criterion of at least 30 laps in 15 min was reached, animals were implanted unilaterally with silicon probes. One probe was implanted in the dorsal hippocampus (HPC) in all the three rats. As to rat 730 and rat 782, another probe was implanted in the medial entorhinal cortex (MEC). The probes used for r730, r782, r829, and r889 were custom single shank, 32 channel probes (NeuroNexus; Ann Arbor, MI) with an area of 177 μ m^2^ and a site spacing of 60 μ m. Rat 1074 received single shank 64 channel probe (L3 series; Cambridge NeuroTech; Cambridge, UK) with an area of 165 μ m^2^ and a site spacing of 50 μ m. Prior to surgery, all probes were cleaned by soaking in a solution of 7% detergent (Contrad 70 Liquid Detergent; Decon Labs; King of Prussia, PA) in deionized water followed by rinsing with deionized water.

### 2.2. Surgical Procedures

All surgical procedures were performed following protocols approved by the Institutional Animal Care and Use Committee at the University of Florida as well as those set forth by the National Institute of Health. Animals were placed in an induction chamber and sedated with 3–5% Isoflurane. After loss of muscle tone, they were moved to a nose cone and the top portion of the head was carefully shaved to avoiding cutting any whiskers. Next, the animal was transferred to the nose cone of the stereotaxic frame, where the head was gently secured with using ear and incisor bars. During this portion and for the remainder of the procedure, anesthesia was maintained using an Isoflurane dose between 1 and 2.5% while periodically monitoring respiration. Body temperature was maintained using an electric heating pad with feedback via rectal thermometer. The eyes were protected by applying ophthalmic ointment and shielding from direct light. Prior to the initial incision, the top of the head was cleaned using several cycles of povidone-Iodine and alcohol. An incision was made starting just behind the eyes and continuing to the back of the skull. The skin was retracted, and blunt dissection was used to expose the surface of the skull. Bleeding was managed using a cautery pen (Bovie Medical; Clearwater, FL). After thoroughly cleaning the skull, measurements from a stereotaxic arm were used to ensure that the skull was leveled. Next, bregma and the electrode implant locations were marked on the skull with the cautery pen for visual reference. A total of seven anchor screws were placed into the skull to serve as attachment points for the headcap. One screw over the cerebellum and one screw over the cortex were attached to wires that would serve as the reference and ground locations, respectively. A small amount of luting cement (C&B Metabond; Parkell Inc; Edgewood, NY) was applied to the screws to provide a foundation for the rest of the headcap. Care was taken to avoid covering bregma and the implant sites. Craniotomies were drilled at the implant sites and the dura was removed, taking care to not damage the cortex. Bleeding was managed using saline irrigation or sterile gauze. Probes targeting the dorsal HPC were implanted at −3.2 mm AP; 1.5 mm ML to bregma; −3.7 mm DV to dura. Coordinates targeting the MEC were −0.5 mm AP to the transverse sinus, 4.6 mm ML to bregma, angled 30° posteriorly, and −5.78 mm DV to dura. After implantation, the craniotomies were sealed with a surgical silicone adhesive (Kwik-Sil; World Precision Instruments; Sarasota, FL). Dental acrylic [Grip Cement, 675571 (powder) and 675572 (solvent); Dentsply Caulk; Milford, DE] was then applied to secure the probes and connectors in place. The ground and reference wires were soldered to the appropriate wires on the probe connectors and the reference wire was isolated using dental acrylic. Lastly, copper mesh was shaped into a small bowl around the headcap to serve as physical protection and secured with dental acrylic. The ground wires were soldered to the copper mesh to minimize the danger of electrostatic discharge. Immediately following the removal of the anesthetic, 10 ml of sterile saline and a dose of 1.0 mg/kg meloxicam (Boehringer Ingelheim Vetmedica, Inc; St. Joseph, MO) were administered subcutaneously. The animals were placed on a heating pad and monitored until fully mobile and capable of eating. Post-surgical care included a second dose of meloxicam 24 h later as well as 5 ml of oral antibiotics (40 mg/ml Sulfamethoxazole and 8 mg/ml Trimethoprim Oral Suspension; Aurobindo Pharma Inc; Dayton, NJ) mixed into their food for 7 days. Animals were monitored for 1 week following surgery to ensure no physical or behavioral abnormalities were observed before testing began.

### 2.3. Euthanasia Electrophysiology

After completing all other behavioral experiments, animals were recorded from for 15 min in the usual resting container to establish a baseline for the LFP data and then received a lethal dose of SomnaSol (390 mg/ml pentobarbital sodium, 50 mg/ml phenytoin sodium; Henry Schein; Melville, NY) injected intraperitoneally. LFP recording continued throughout the injection and for 10–15 min after the animal no longer exhibited a nociceptive withdrawal reflex. The animal was then immediately perfused with 4% paraformaldehyde, and the brain extracted and prepared for histology to verify electrode locations.

### 2.4. Data Process and Spectral Analysis

The LFP data were analyzed in MATLAB (The MathWorks, Natick, MA) using custom-written code as well as code imported from the HOSAtoolbox (Swami et al., 2000). Raw LFP records sampled at 24 kHz (Tucker-Davis system) were low-pass filtered down to 1 kHz. The spectrogram were calculated based on discrete Fourier transform with window length of 1 s and 50% overlap. The power correlograms for each evolution stage were obtained by estimating the correlation coefficients between all the frequency pairs in the result of spectrogram. The power spectra during the euthanasia were estimated for every 100 s. Within each LFP interval, the power spectrum was obtained via the standard Welch's method (Welch, 1967) with window length of 1 s and 50% overlap. The power law exponential was obtained by linearly fit the log-log power spectrum in the frequency range from 20 to 80 Hz to diminish the influence of EMG noise.

The coherence between two time series is the modulus of their cross-spectrum normalized by their power spectra. The cross-spectrum and power spectra were estimated with window length of 1 s and 50% overlap. To demonstrate the time evolution of coherence, a sliding window with a length of 20 s and a step increment of 5 s was applied. In each window, the coherence between two time series in frequency range from 2 to 128 Hz were estimated.

The asymmetry and skewness of LFP were obtained from the bispectral analysis with a window length of 1 s and 50% overlap. The bispectrum (the Fourier transform of the third-order cumulant) has been thoroughly reviewed in terms of both statistical and mathematical background (Harris, 1967) as well as its application to non-linear wave interaction (Kim and Powers, 1979). In the field of neuroscience, bispectral analysis was used to quantify the degree of phase coupling between the frequencies of the LFP, whereas the bicoherence quantifies the degree of cross-frequency coupling independent from the amplitude (Barnett et al., 1971; Ning and Bronzino, 1989; Sigl and Chamoun, 1994; Bullock et al., 1997; Muthuswamy et al., 1999; Hagihira et al., 2001; Li et al., 2009; Sheremet et al., 2016; Wang et al., 2017; Avarvand et al., 2018). The cross-bispectrum analysis is similar with the bispectrum analysis but the frequency components are from two time series (Lii and Helland, 1981; Sheremet et al., 2020). In our study, as the lengths of stage 2 differ across regions, the cross-bicoherence between HPC and MEC were estimated over the shorter stage length.

The decay rate of each frequency component was derived from the power time series. Power time series of frequency component ω was defined as the variance of filtered LFP in the frequency band [ω − 0.5Hz, ω + 0.5Hz]. The power time series was calculated with a window length of 10 s and a step increment of 2 s (**Figure 3C**). The decay/grow constant was defined as the ratio of the time derivative of the power time series to the power time series itself. The obtained result was smoothed with the time window of 20 s to eliminate fast power oscillations, and was plotted every 5 s. The transition period from the first to the second stage is defined as the moment with the fastest averaged decay rate over 100 Hz. The transition period from the second to the third stage is defined as the moment with the fastest averaged decay rate from 4 to 60 Hz.

## 3. Results

### 3.1. Three Stages During Spectrum Degradation

After the injection of SomnaSol, LFP exhibited three distinguishable stages with two rapid transition periods between these stages in all the animals.

#### 3.1.1. Pre-effective Stage

**Figure 1:**
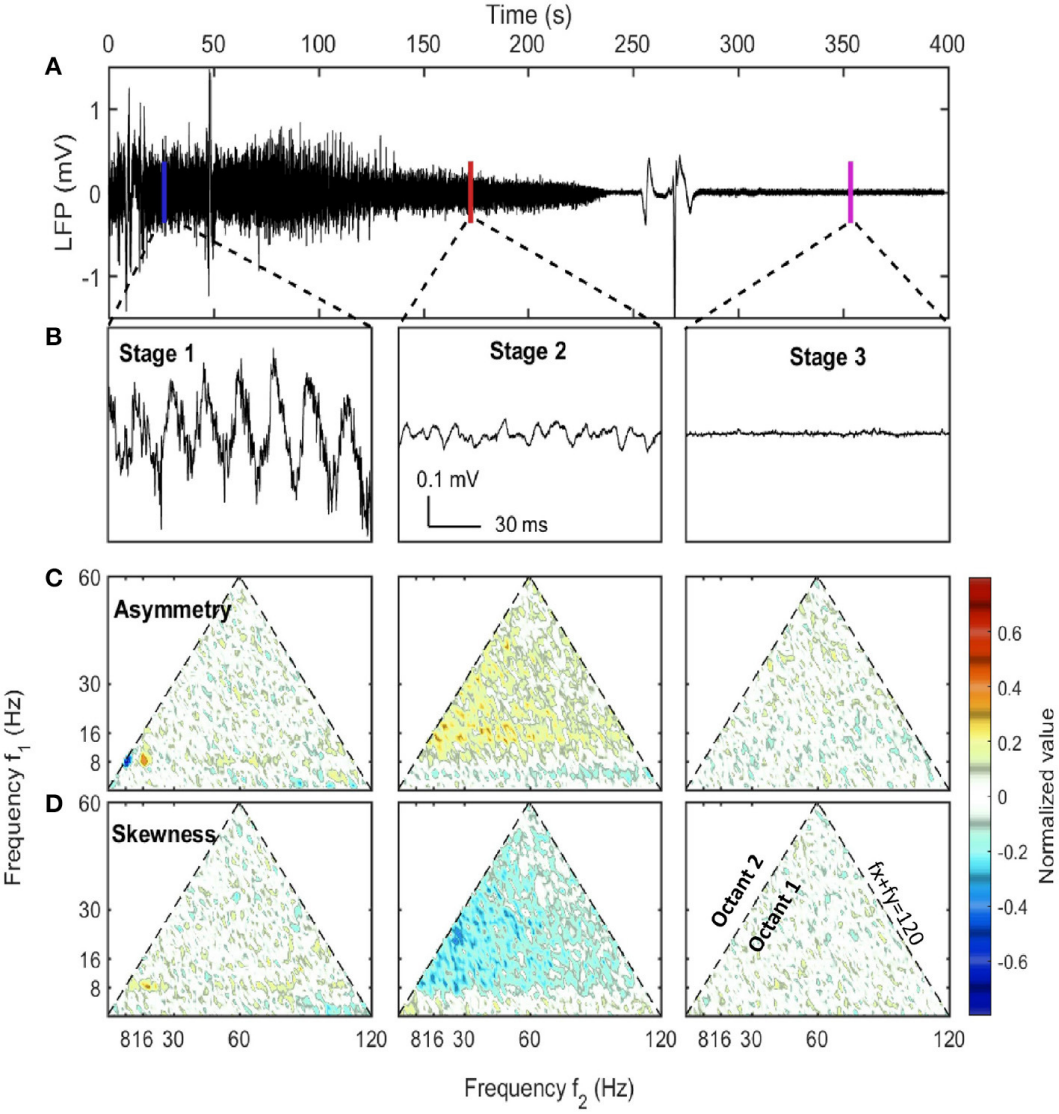
LFP examples at different stages along with their asymmetry and skewness. **(A)** The raw LFP trace recorded in CA1 pyramidal layer after the injection (*t* = 0) which showed the overall neural activity evolution during euthanasia. **(B)** LFP samples of 1 s selected from three stages. The corresponding windows were indicated in **(A)**. **(C,D)** The asymmetry and the skewness of LFP at three stages obtained from bispectral analysis (Haubrich and MacKenzie, 1965; Masuda and Kuo, 1981; Sheremet et al., 2016). Both the horizontal axis and the vertical axis represent frequency. Each point in the plot was the asymmetry or skewness estimated for the frequency triad (*f*_*x*_, *f*_*y*_, *f*_*x*_ + *f*_*y*_). Only the first octant (*f*_*x*_ ≥ 0, *f*_*y*_ ≥ 0, *f*_*x*_ ≥ *f*_*y*_) is presented as it contained all the non-redundant information due to the symmetry of bispectrum. The first octant is bounded by the dashed line *f*_*x*_ + *f*_*y*_ = 120 Hz indicating the upper bound of frequency range of interest. An oscillation has asymmetry if the wave peak or trough doesn't stand at the center of adjacent zero-across points. An oscillation has skewness if the wave height distribution is not symmetric about its mean value. Both asymmetry and skewness indicate cross-frequency coupling. Data from rat 782.

**Figure 2:**
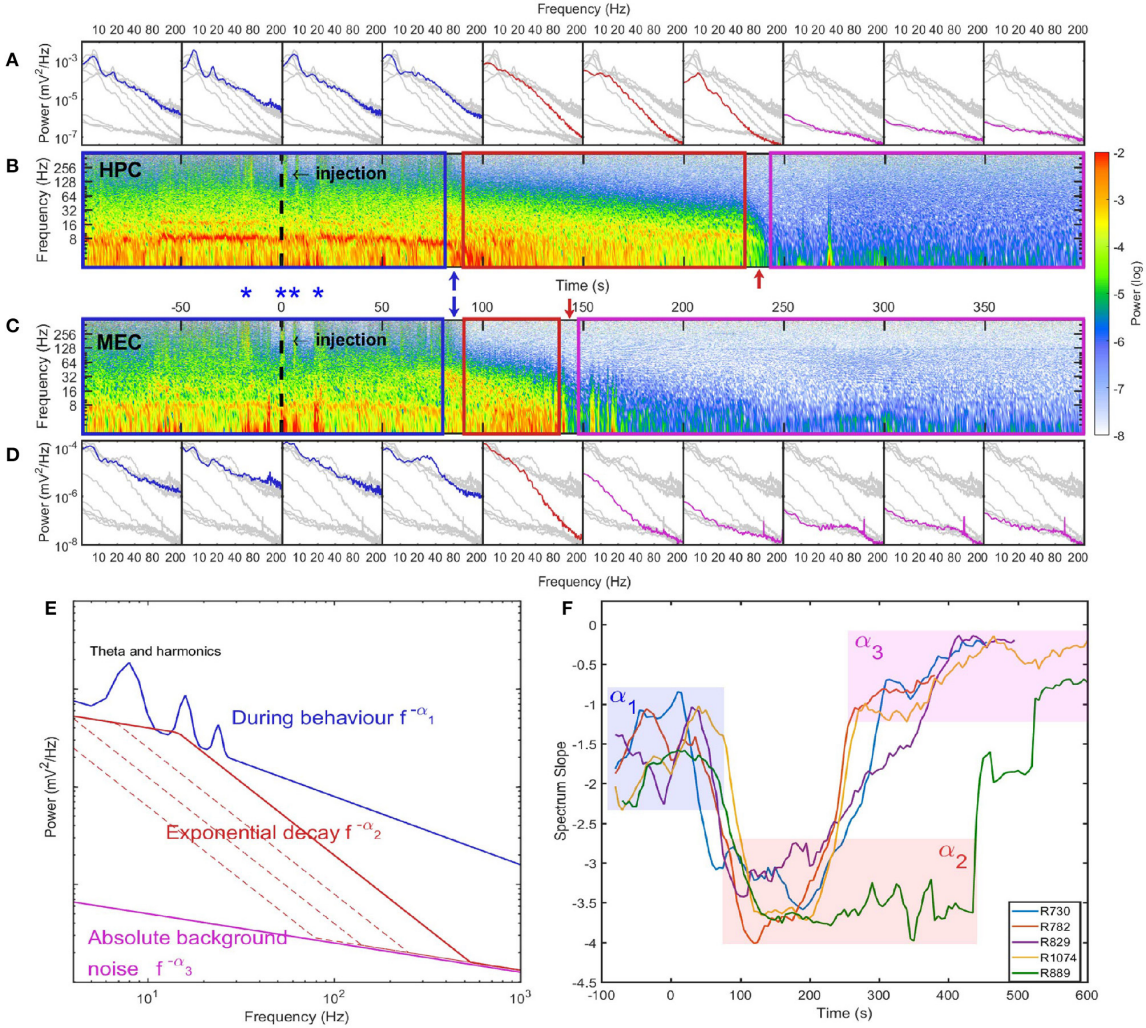
Spectrum evolution during barbiturate overdose euthanasia. **(A)** The development of spectra estimated every 50 s. Spectra estimated within other time intervals were indicated as gray lines for comparison. **(B)** The spectrogram of hippocampal LFP where the injection time was marked as a dashed line at 0 s. The power in spectrogram was normalized by the maximum power during the euthanasia process. Based on the spectrogram and the development of power spectrum, we identified three stages during euthanasia: stage 1 was the pre-effective stage which included the pre-injection period and a short period after injection. This demarcated in the spectrogram as a blue box and in spectra, blue lines; stage 2 was the quasi-stationary decay stage marked as a red box in spectrogram and red lines in spectra; stage 3 was the quasi-white noise stage marked as a magenta box in spectrogram and magenta lines in spectra. Between these stages there were two rapid transition periods marked with blue and red arrows. **(C)** The spectrogram of LFP recorded in MEC. During stage 1, there were instances with strong high-frequency power (>128 Hz) both can be observed in HPC and MEC. Four of these instances were marked as blue asterisks. Given the consistency across regions, these high frequency bursts are most-likely related to sodium pentobarbital related muscle contractions (EMG artifact, see Supplementary Figure 3). **(D)** The development of spectra in MEC. Data from rat 782. **(E)** Cartoon illustrate the spectrum evolution during euthanasia. The spectra from the first to the third stages were marked as blue, red and magenta lines. The power spectra exhibits different slopes across stages. **(F)** Power-law exponents evolution of five rats estimated with window length of 40 s and time increment of 5 s. The spectrum slopes were estimated for the frequency range from 20 to 80 Hz in the log-log plot. Three stages can be identified with α_1_ ≈ 1.5, α_2_ ≈ 3.5, and α_3_ ≈ 0.5. Note that rat 889 had longer stage 2 compared with other rats (see Supplementary Figure 6).

**Figure 3:**
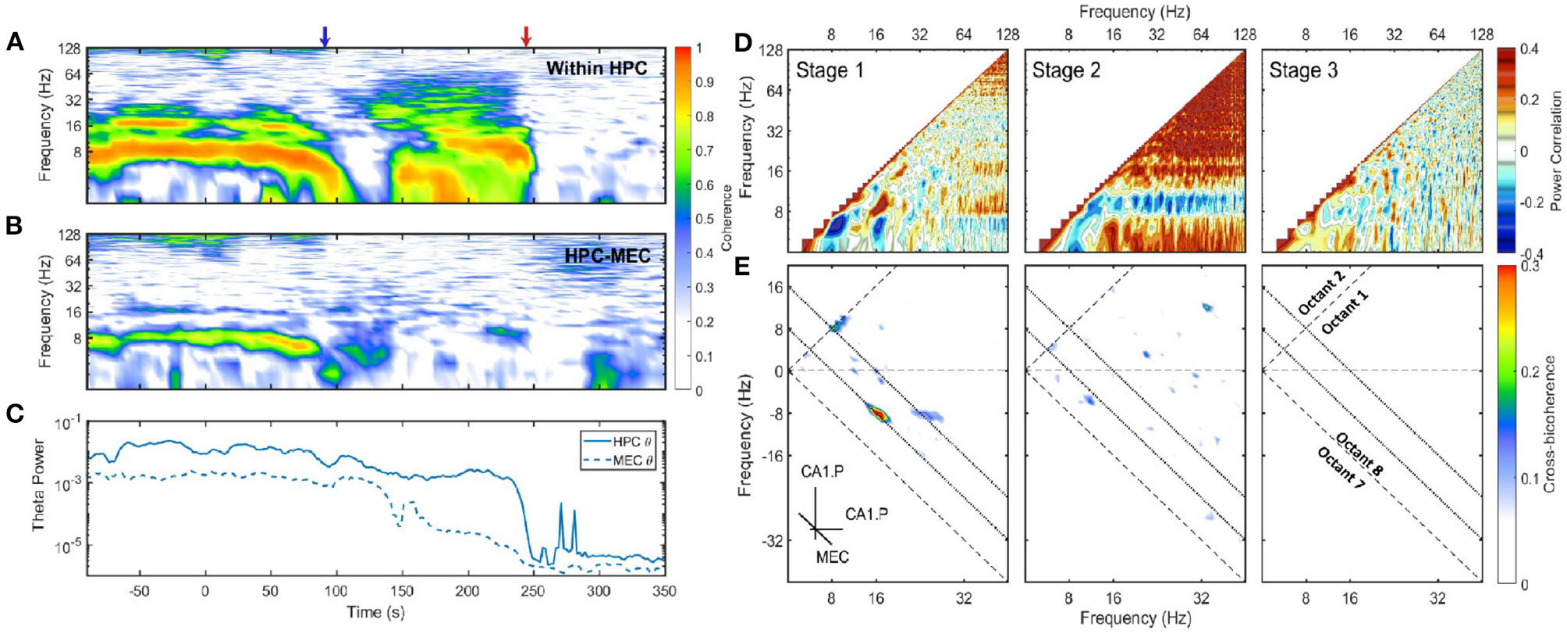
**(A)** Coherence between CA1.Pyr and LM in HPC. The x-axis was the time and the y-axis was the frequency. Two transition periods were marked with blue and red arrows in the plot. Coherence is the modulus of the normalized cross-spectrum with a value between 0 and 1. High coherence indicates there is a consistent phase difference between two time series at that frequency. In terms of spectral analysis of LFP, high coherence within a narrow frequency band is a sign of the existence of oscillatory rhythm, and there is a phase difference either because the rhythm is a traveling wave or due to the bipolar nature of neurons. **(B)** The development of coherence between HPC and MEC. **(C)** The energy evolution of theta band in HPC and MEC, the variances were calculated with a window length of 10 s and a step increment of 2 s. **(D)** Average auto-correlation coefficients of Fourier transform estimated at three stages. As the auto-correlations are symmetric, only one half was presented. Positive correlation indicates the power of those two frequency components tend to grow or decay simultaneously, while negative correlation demonstrates that the power in some frequencies is lost as others increase (see Masimore et al., 2004, 2005). During stage 1, the positive correlation can be identified in three regions: The correlation between theta and theta harmonics represented by significant dots with frequency under 32 Hz; The correlation between gamma (>60 Hz) and theta (or theta harmonics) represented by horizontal strips. During stage 2, the power of 10 Hz frequency components was negatively correlated with other frequency components, while all other frequency components had positive power correlation. **(E)** Cross-bicoherence between HPC and MEC. Each point in the plot represented the cross-frequency coupling of the frequency triad (*f*_*x*_, *f*_*y*_, *f*_*x*_ + *f*_*y*_) where *f*_*x*_ and *f*_*y*_ were frequency components in HPC and *f*_*x*_ + *f*_*y*_ was frequency component in MEC. Octant 1 and Octant 8 contained all the non-redundant information. Two inclined dotted lines indicated 8 and 16 Hz in MEC. During the stage 1, there were significant regions representing cross-region cross-frequency coupling of theta and its harmonics between HPC and MEC [(8, 8, 16) Hz, (16, −8, 8) Hz and (24, −8, 16)Hz]. Data from r782.

The first stage (marked as blue box in **Figure 2**) was described as the pre-effective stage,or stage 1. This stage was indistinguishable from baseline (pre-injection).Thus in Figures 1, **3**, both the pre-injection (t < 0) and the pre-effective stages were marked as stage 1. In this stage, LFP was dependent on the behaving state of the rat where there were strong theta and gamma rhythms at high running velocity (Whishaw and Vanderwolf, 1973; Morris and Hagan, 1983; Chen et al., 2011; Ahmed and Mehta, 2012; Kemere et al., 2013; Zheng et al., 2015; Sheremet et al., 2019a). A sample of hippocampal LFP from stage 1 (Figure 1B) reveals that theta can express significant deviations from a sinusoid (Buzsaki et al., 1983; Terrazas et al., 2005). This non-sinusoidal waveform is related to high order theta harmonics due to the non-linearity of hippocampal LFP (Scheffer-Teixeira and Tort, 2016; Sheremet et al., 2016; Zhou et al., 2019). In statistical analysis, the lowest order non-linear character of the system can be described by bispectrum (Hasselmann et al., 1963). The real and imaginary part of the bispectrum characterizes the skewness (an example being a cnoidal wave) and the asymmetry (“sawtooth” shaped wave) of the distribution. The sawtooth aspect of the theta wave, with steep wave front (from trough to peak), corresponded to the negative asymmetry (Figure 1C) at the [8, 8, 16 Hz] frequency triad (8 Hz at x-axis, 8 Hz at y-axis and their sum 16 Hz). Apart from that strong negative asymmetry region, the [8, 16, 24 Hz] frequency triad exhibited strong positive asymmetry and supported the existence of third-order theta harmonic (Schomburg et al., 2014; Zhou et al., 2019; Cowen et al., 2020).

In the spectrogram of hippocampal LFP (Figure 1B) during stage 1, there was a strong theta oscillation along with intermittent but distinguishable second and third harmonics. Apart from high power activities in theta and its harmonics bands, intermittent high-frequency events were identified by looking at spectrogram at frequency range over 128 Hz (marked as blue asterisks in Figures 2B,C). Although the frequency range overlaps with *epsilon* rhythms described by others Canolty et al., 2006; Freeman, 2007; Sullivan et al., 2011; Belluscio et al., 2012, we make the conservative interpretations that is it caused by abnormal muscle contractions. High-frequency neural events tend to be local as they are supported by interactions within a specific brain region (Buzsaki and Draguhn, 2004; Buzsaki, 2006; Sheremet et al., 2019a), but the high-frequency intermittent bursts during stage 1 were highly coherent and had almost zero phase lag between HPC and MEC regions (Supplementary Figure 3). Therefore, the intermittent high-frequency bursts are plausibly related to muscle activities (EMG noise; Muthukumaraswamy, 2013; Nottage and Horder, 2015) as pentobarbital is known to include abnormal contractile activity (Nayler and Szeto, 1972; Khan, 1980; Taylor et al., 1984; Eikermann et al., 2009). This activity subsided after stage 1 due to pentobarbital sodium injection (Altura and Altura, 1975).

#### 3.1.2. Quasi-Stationary Decay Stage

The second stage (marked as red box in Figure 2) was described as the quasi-stationary decay stage, or stage 2, after the sudden disappearance of theta and >128 Hz bursts (at 100 ± 33 s after SomnaSol administration). The hippocampal LFP in this quasi-stationary stage exhibited slow power decay over a period of ~100 s without obvious intermittent structures (Figure 1A). Within a narrow time frame, the LFP appeared to be nearly stationary, although of lower amplitude compared with stage 1. Traditional 8 Hz theta activity was no longer evident via visual inspection. Rather, a surge in lower frequency band activity (1–4 Hz) occurred and this event has been reported previously (Schramm et al., 2020). As the power in this lower frequency, 1–4 Hz activity dissipated, a 10 Hz oscillation became prominent, characterized by a wide-flat peak and narrow-deep trough (Figure 1B). This waveform expressed a negative skewness in the frequency region from 10 to 30 Hz via bispectrum analysis (Figure 1D).

The spectrogram of LFP during stage 2 was consistent with observations of the time-series. There were no intermittent high-frequency structures (EMG artifacts), but there was the development of a 10 Hz oscillation at the end of stage 2. In terms of the spectrum evolution, except for the frequency components <10 Hz, there was a structured power decay over a wide frequency band.The power-law distribution persisted over the entire stage with a slope of −α_2_ ≈ −3.3, but with a decaying total variance (Figures 2E,F). There was a progressive recession of power, where the power in the 20–120 Hz decreased at the same logarithmic rate. This degradation is evident in the straight power contour lines in the spectrogram plot (contour lines were not directly plotted but can be identified from the transition of colors) (Figure 2B). As a result, during stage 2 the spectrum experienced a “parallel” evolution that can be interpreted as the entire spectrum having a decay in power along with a shift toward lower frequency. By comparing across animals, we observed a “parallel” spectrum evolution in HPC and MEC regions (Figure 2 and Supplementary Figures 4–7). However, although the onsets of stage 2 were synchronized, their lengths varied between HPC and MEC. In the HPC region, the power-law spectrum kept evolving after the LFP spectrum in MEC had collapsed to a low power-containing state.

#### 3.1.3. Collapse Stage

In the HPC region, the LFP collapsed at 320 ± 101 seconds after SomnaSol administration, marking the spectral transition into stage 3, or collapse stage (marked as a magenta box in Figure 2). Oscillation amplitude was small (Figure 1B) with occasional large “LFP-spikes” (Figures 1A, 2B). This oscillation has been described previously as the “wave of death” (WoD; Kaminogo et al., 1998; Van Rijn et al., 2011; Zandt et al., 2011), which is proposed to reflect the massive and simultaneous depolarization of a large number of neurons. This phenomenon most-likely shares a high degree of similarity with cortical spreading depression (Dreier and Reiffurth, 2015), accounting for why the event was not highly correlated between brain regions (Figures 2B,C). Pani et al. (2018) reported this brain activity could persist for about 120 min after cardiac arrest, maintained by a slow spreading depolarization caused by irreversible degenerative processes at the cellular level. Apart from the transient spikes, LFP in stage 3 did not show significant asymmetry or skewness in the bispectral analysis (Figures 1C,D). The power spectrum during stage 3 was flat with a slope of −α_3_ ≈ −0.75 and represented the least power containing state of our LFP measurement (Figures 1E,F). Given the low power level during stage 3, it is possible that the spectrum reflected more of the electro-thermal noise in the physical recording environment surrounding the data acquisition system than neural activities.

### 3.2. Degradation of Theta After the First Stage

In the previous section, we stated the stage 1 of spectrum degradation ended with the erosion of observable theta and the disappearance of the intermittent high-frequency activity. Comparing HPC and MEC spectrograms, we observed that the transition from stage 1 to stage 2 was synchronized across these regions (Figure 2, Supplementary Figures 4B,C). Although the typical theta oscillation along with its harmonics was no longer visually observable in the raw LFP after stage 1 (Figures 2B,C), the power in theta band (6–10 Hz) only exhibited a limited decrease from stage 1 to stage 2 in the HPC and MEC regions (Figure 3C and Supplementary Figures 8–11). Therefore, to determine if the 6–10 Hz frequency component is a degenerate form of theta or due to a different mechanism, we sought to determine if the band shared features generally associated with theta.

A linear and non-linear spectral analysis was conducted to investigate the coupling in theta band within HPC and across regions. During stage 1, strong coherence existed at theta and theta harmonics frequency range across HPC layers (Figure 3A). The high coherence was consistent with the observation that the hippocampal LFP was dominated by theta waves, and the theta oscillation experienced a phase reversal at hippocampal fissure (Winson, 1974). The coherence at theta rhythm can also be observed between HPC and MEC regions, which was expected given the strong reciprocal connections between these structures and the observation of traveling theta waves in both regions (Lubenov and Siapas, 2009; Hernández-Pérez et al., 2020). To identify the fundamental frequencies of the LFP and determine any potential interactions across different oscillatory bands, a power correlation analysis (Masimore et al., 2004, 2005) was conducted. The analysis revealed that during stage 1, the power of theta, the power of theta harmonics, and the gamma rhythm (60–120 Hz) were all positively correlated (Figure 3D). In terms of non-linear cross-frequency coupling. the non-linearity of theta can be investigated through the use of the bispectrum, expressed as a significant region at the frequency triad (8, 8, 16) Hz (Figure 1). The cross-frequency coupling existed not only within the hippocampal region, but also between HPC and MEC regions. Figure 3E showed the cross-region cross-frequency coupling for the frequency triad (*f*_*x*_, *f*_*y*_, *f*_*x*_ + *f*_*y*_) where *f*_*x*_, *f*_*y*_ belonged to CA1.Pyr and *f*_*x*+*y*_ belonged to MEC. The cross-bispectral analysis revealed that theta and theta harmonics were cross-frequency coupled across HPC and MEC regions during stage 1. To summarize, theta rhythm in stage 1 had the following features: (1) The oscillations at theta range were coherent within HPC, and between HPC and MEC. (2) The power of theta is positively correlated with power of gamma. (3) When the power of theta was strong, theta was phase-coupled with high order theta harmonics.

After stage 1, there was considerable power persisting in the 6-10 Hz frequency band at the end of stage 2. Specifically, there was high coherence around 10 Hz within the hippocampus (Figure 3A). However, the across region coherence was weak (Figure 3B). Moreover, as the majority of frequencies degraded together, the power correlation during the second stage- except for the 10 Hz oscillation- were positive. That was consistent with the “parallel” spectrum degradation where all the frequency components experienced power decay. The 10 Hz oscillation, however, had a negative power correlation due to the power increase at the end of stage 2 (Figure 3D). The cross-bicoherence also showed that the cross-frequency coupling between HPC and MEC was weak during stage 2 compared with stage 1 (Figure 3E). Although the oscillation had a frequency close to theta and high coherence within HPC region, it was not coherent between HPC and MEC, nor correlated with other frequency bands. Therefore, it is most-likely distinct from theta as it does not engage a large population of neurons across brain regions, but is perhaps related to a local hippocampal network dynamic (e.g., O'Keefe and Recce, 1993).

### 3.3. Uniform Exponential Power Decay in the Second Stage

**Figure 4:**
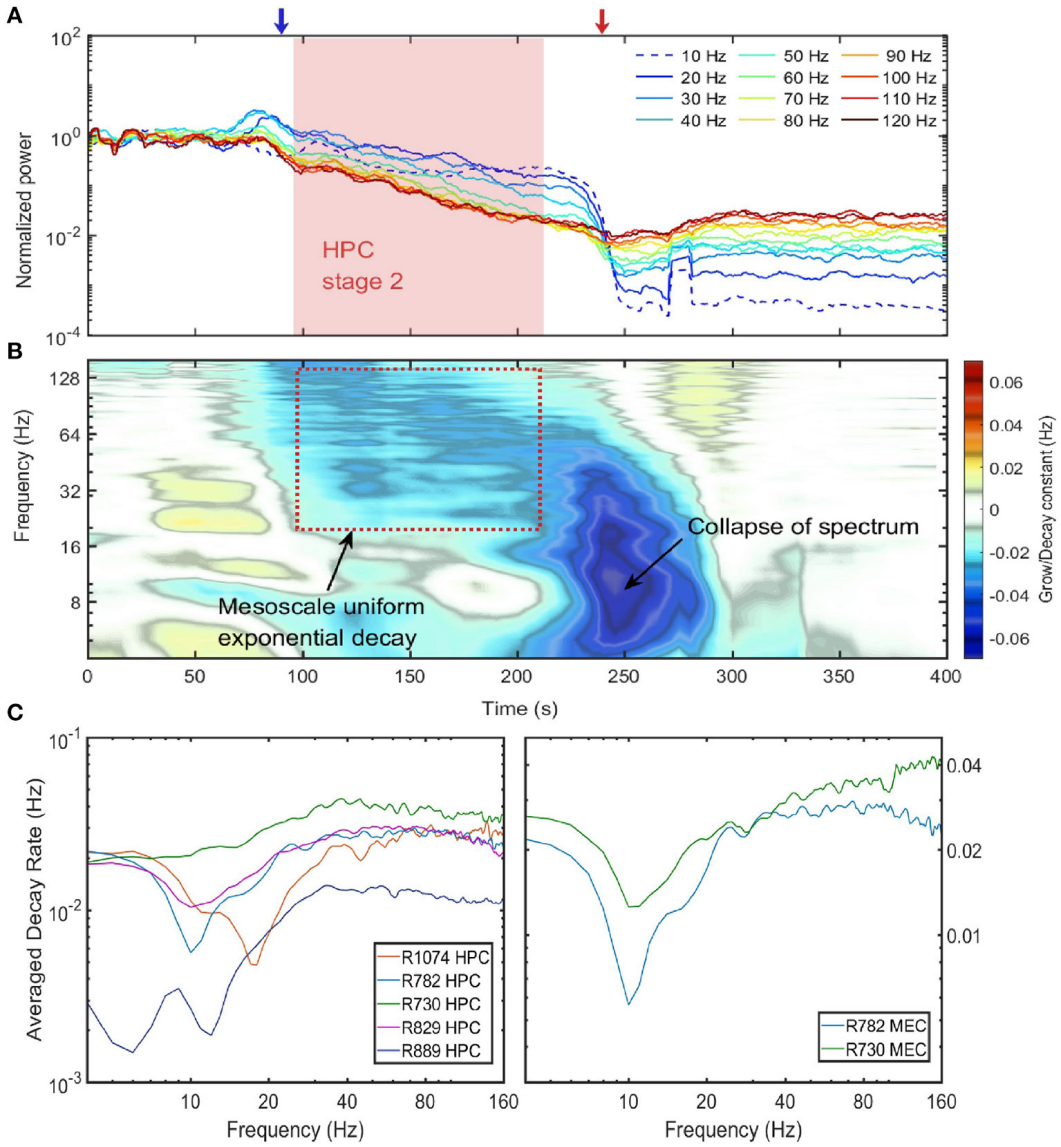
Power decay of frequency components over the euthanasia process. **(A)** The power evolution for frequency component from 10 to 120 Hz. Each line corresponded to the power decay of one frequency component. Low frequency components were indicated by cold colors and high-frequency components by warm colors. Note the power were normalized by their value at the time of injection (*t* = 0), and the 10 Hz frequency component experienced a growth at the end of stage 2. Data from rat 782. **(B)** The percentage power change rates of different frequency components over the entire spectrum degradation process. To obtain the percentage power change rate, the power time series for each frequency component was first estimated and the percentage power change rate was defined as the ratio of the time derivative of power time series to the power time series. If a frequency component experiences a exponential decay or grow *e*^α*t*^, the percentage power change rate will be a constant with value α. Data from rat 782. **(C)** The decay constant vs. the frequency during the exponential decay stage. The left panel were the decay constants in HPC among rats and the right panel were decay constants in MEC. The decay constant were estimated by averaging over the entire stage 2 period. Note that the overall decay rate of rat 889 was smaller compared with other rats because rat 889 had a longer stage 2 (see Supplementary Figure 6).

In the previous section we have shown that during stage 2, except for the 10 Hz oscillation, the power of all the frequency components were positively correlated in that their power receded together. In this section, we investigated how power of different frequency components evolved during the entire euthanasia process (Figure 4A). According to the power evolution plot there are two periods of rapid change: (1) In the transition from stage 1 to stage 2, there was a marked divergence between low and high frequencies, potentially carried by the development of 10 Hz power and the loss of power in higher frequencies. (2) From stage 2 to stage 3, low frequency components experienced another rapid decay because of the collapse of power-law spectrum (red arrow in Figure 4A). Between these two transition periods, the power evolution of frequency components from 20 to 120 Hz can be approximated as straight lines. In the semi-log plot the straight line evolution can be interpreted as exponential decay (stage 2 indicated as red box in Figures 4A,B). The slopes of these power lines reflected the decay rates of corresponding frequency components, and during stage 2 the power evolution were almost parallel which indicated that frequency components shared a similar decaying rate.

The decaying rate (in the unit of Hz) can be quantified by differentiating power time-series with respect to time, and normalizing the time derivative by the power (Figure 4B). During stage 2 of spectrum degradation, the frequency components over 20 Hz decayed at similar rate with a decay constant around 0.03 Hz. This exponential decay of high-frequency components lasted shorter amounts of time as their power reached the low background energy plateau and experienced limited power decay afterwards. Stage 2 ended with a rapid decay of low frequency components with decaying rat of 0.06 Hz corresponding to the collapse of the power-law spectrum.

Similar rates of power decay during stage 2 were observed across animals and regions (Figure 4C). The decay rates of frequency components lower than 20 Hz were small due to the development of a 10 Hz rhythm at the end of stage 2. The spectrogram in Figure 2 indicated that the 10 Hz frequency was a degraded form of a 20 Hz rhythm presented earlier in the degradation process. In the spectra evolution (Figure 2A and Supplementary Figures 4–7) the 10 to 20 Hz oscillation acted as a spectrum front, but the reason of its generation and development is unknown. Apart from that, other frequency components had a exponential decay with decay constant at the magnitude of 0.03 Hz in HPC in MEC. One exception is rat 889 which had a longer stage 2 and the decay rates were significantly lower than other rats.

**Figure 5:**
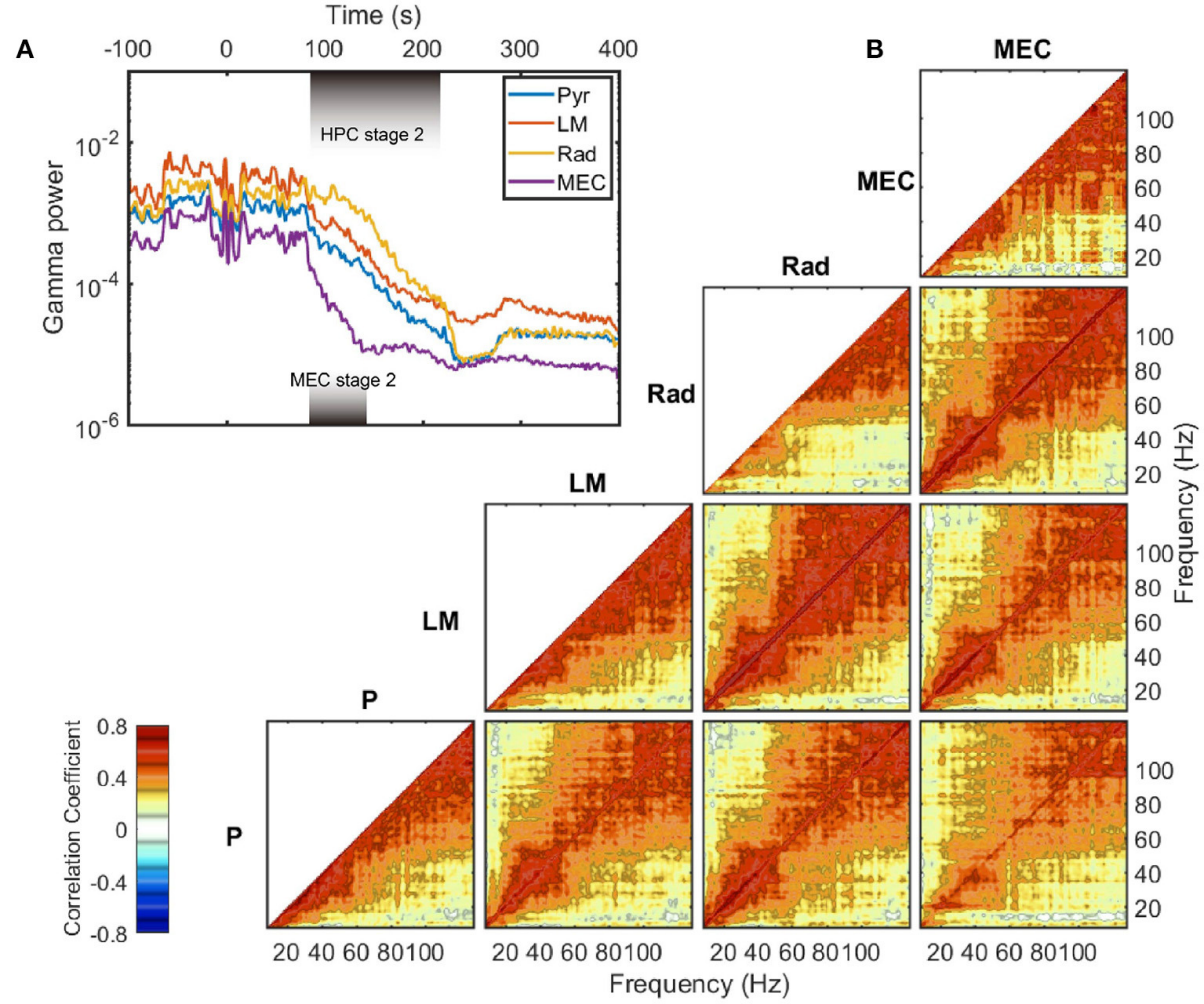
Gamma decay during stage 2. **(A)** The evolution of LFP variance band-passed in gamma range (60–120 Hz) in different layers. The durations of stage 2 are different in HPC and MEC regions, and are marked with shaded rectangles in the plot. Data from rat 782. **(B)** Averaged auto- and cross correlation coefficient of decay rate during stage 2. The decay rate (defined as in Figure 4B) of each frequency component was computed and treated as a time series. Then the correlation coefficients were estimated for time series pairs from either the same layer or different layers. Within a region, the correlation of a frequency's decay rate with itself is equal to one. Furthermore, as the autocorrelations are symmetric, only one half is presented. The cross-correlation of decay rate, however, can vary across unity and is not necessarily symmetric. For instance, the bottom right panel represents the correlation of the decay rates in pyramidal (y axis) to that in MEC layer (x axis). Note that for the MEC-related panels (the rightmost column), the data were averaged between rat 730 and rat 782, and the decay rates were estimated over the MEC stage 2 period. For other panels, the results were averaged across all the five rats. P, pyramidal layer; Rad, radiatum; LM, lacunosum-moleculare; MEC, medial entorhinal cortex.

The exponential decay occurred within the gamma range (Bragin et al., 1995; Chorbak and Buzsaki, 1998), defined here as 60–120 Hz based on the power-power couplings observed in Figure 3D and based on prior publications (Sheremet et al., 2019a; Zhou et al., 2019). As the gamma oscillations are short-lived and typically emerge from the coordinated interaction of excitation and inhibition (Buzsaki and Wang, 2012), the patterns of gamma rhythm is closely related to local circuit connections and thus have layer specification. To investigate whether the development of gamma rhythm during barbiturate overdose euthanasia is dependent on layers, the evolution of gamma power in layer pyramidal layer (Pyr), radiatum (Rad), lacunosum-moleculare (LM), and MEC layer were plotted (Figure 5A). The hippocampal electrode location was confirmed by current source density (CSD) analysis of hippocampal sharp-wave ripple events (Supplementary Figure 2). During stage 2, MEC gamma rhythm experienced a faster decay compared with that in HPC strata, which was reflected as a steeper slope in the semi-log gamma power plot. Within the HPC region, however, the degradation of the gamma rhythm did not exhibit strong layer dependence. The gamma power curves were almost parallel during stage 2, with the initial gamma power being the primary difference. The power decay rates of frequency components in gamma range also exhibits strong correlation within and across layers (Figure 5B). Apart from the low frequency range which was influenced by the development of 10 Hz oscillation during stage 2, most of the frequency pairs had a correlation coefficient in decay rate over 0.4, and the correlation coefficients grow as approaching the diagonal. The strong correlated decay rates across frequency pairs implied that during stage 2, the frequency components in gamma band experienced a uniform power deprivation. As the cross layer decay rate correlation had similar magnitude with same layer correlation, we reported that no significant layer dependence of gamma evolution was observed during stage 2 of barbiturate overdose euthanasia.

## 4. Discussion

The current manuscript investigated the degradation of the hippocampal power spectral density over the course of euthanasia. Perhaps the most outstanding result is the magnitude of power loss across all frequency bands. Comparisons of power spectral densities between sleep and wake states (e.g., Figure 4 of Buzsáki et al., 2003; Supplementary Figure 1) reveal that the overall “1/f” is well-preserved even in quiescence. The dramatic collapse over euthanasia suggests that the brain is in a persistent state of near-maximal activity, a conclusion arrived upon in perhaps the earliest characterization of the 1/f power spectral density: “*Our theory outlined above claims that the sum of energy is held at the maximum even in the passive state of consciousness*…” (Motokawa, 1949). This is to emphasize that, as the LFP is primarily shaped by synaptic transmembrane current (Buzsaki et al., 2012), the normal functioning brain is constantly maintaining baseline activity far from equilibrium. And although the slope of the spectra has been known to change as a function of running speed (Sheremet et al., 2019b), the parametric space in which the 1/f slope changes is small relative to power in specific frequency bands, such as theta. Therefore, examining the LFP over the course of euthanasia offers a larger parametric space to explore the mechanisms that support the 1/f spectra.

We observed three states of spectral degradation. Stage 1, or pre-effective stage, can be described as a typical “active,” theta-dominate state where the 7–10 Hz rhythm is readily identifiable in the LFP and the power spectrum. In stage 2, or the quasi-stationary decay stage, the theta peak above the 1/f slope degraded. This was associated with a reduced theta coherence between the medial entorhinal cortex and hippocampus. This suggests that, in the early part of stage two, the ability of the medial entorhinal cortex and hippocampus to interact is impaired but not absent. As stage two progressed, the spectrum displayed a “parallel” degradation that can be interpreted as the entire spectrum having a decay in power along with a shift toward lower frequency. Interestingly, the erosion in higher frequency bands (including the 60–120 Hz gamma band) was accompanied by the appearance of a 10 Hz peak in the power spectral density. The duration of stage 2 persisted longer in the hippocampus relative to the medial entorhinal cortex (which did not exhibit a 10 Hz peak). The end of stage 2 and start of stage 3 was marked by a complete collapse of the power in all spectral bands, with low activity in nearly all bands and occasional large spikes that plausibly related to spreading depolarization (Pani et al., 2018). Finally, the activity reached a minimum, credibly being the electro-thermal noise in the physical recording environment surrounding the data acquisition system.

As it would be a rare instance for a single experiment to comprise a full test of any hypothesis, it is first necessary to discuss the limitations of the current study before making theoretical extensions. The most-outstanding limitation of the study was the use of the pharmacological agent to induce euthanasia. The action of SomnaSol is systemic, making it difficult to infer the spectral decline mechanism. We cannot expressly state that the lack of perfusion, the action of the barbiturate on the GABAergic system, the combination thereof, etc., is exclusively responsible for the recession in power seen in specific frequency bands. Furthermore, pentobarbital is known to augment the contractile activity of muscle fibers and reduce the cells' ability to maintain appropriate calcium homeostasis, thus initiating a contracted state (Nayler and Szeto, 1972; Khan, 1980; Taylor et al., 1984; Eikermann et al., 2009). We observed high-frequency bursts above 128 Hz, most-likely being an EMG artifact as the rhythm is phase-locked across regions (see Supplementary Figure 9). Unfortunately, this precludes the ability to either examine high-frequency rhythms or action potential activity of the neurons. Moreover, the large LFP spikes between stages 2 and 3 could be a filtered version of cortical spreading depression due to AC-coupled amplifiers. Future studies should address these limitations by using alternative forms of euthanasia, attempt to minimize EMG artifacts, and implement a DC-coupled recording to explore the cortical spreading depression.

Nevertheless, the use of SomnaSol resulted in a total spectral collapse, with a recession in power from high to low frequencies. We interpret these results from the perspective of the energy cascade hypothesis, where low frequency, high power rhythms are a function of large-scale activity extending beyond a single brain region and high-frequency, low amplitude oscillations are small-scale interactions (Buzsaki and Draguhn, 2004; Buzsaki, 2006; Sheremet et al., 2019a). Relative to the more extensive networks, smaller networks are less fault-tolerant as they are fewer in number and have fewer supporting synaptic connections. Thus, as there is less degeneracy/redundancy in small networks, compromising the function of a few neurons would be more catastrophic. The large number of neurons and extensive connectivity in a network supporting slower frequency rhythms would be more fault-tolerant, allowing it the ability to suffer a more significant loss prior to collapsing. This idea is supported by the observation of a progressive recession of power throughout stage 2. As more neurons succumb, there is a progressive loss of power from high to low frequencies that continues until the final remnants of rhythmic activity is supported by the most robust network.

At the end of stage 2, the erosion of high-frequency power appears to temporarily stop at ~10 Hz in the hippocampus. Theoretically, this “last stop” in rhythmicity may be a function of the dense anatomical connectivity within the hippocampus (Lorente de No, 1938), with a frequency that reflects the intrinsic membrane resonance properties of the pyramidal cells and a subpopulation of interneurons (O'Keefe and Recce, 1993; Kamondi et al., 1998; Yamaguchi and McNaughton, 1998; Bose et al., 2000; Magee, 2001; Lengyel et al., 2003; Maurer et al., 2005, 2006; Geisler et al., 2007). Finally, when either there is a critical loss of hippocampal neuron function or the excitatory input into the hippocampus declines to the point that it is no longer able to excite a sufficient proportion of the hippocampal population, there is a complete collapse of the spectrum to quiescent levels. Notably, the medial entorhinal cortex exhibited a collapse in the faster rhythms prior to the collapse of the same frequencies across the hippocampal layers. This suggests that the mechanisms that support the higher-frequency rhythms in the hippocampus, such as gamma, are not necessarily relayed into the hippocampus but are most-likely generated locally (Buzsaki and Wang, 2012). As interneurons are prevalent across hippocampal layers (Klausberger and Somogyi, 2008), it is tenable that GABAergic circuits support the gamma rhythm observed across layers, with oriens lacunosum moleculare interneurons plausibly playing a major role in shaping the high frequency power in the lacunosum moleculare layer of CA1.

Up to this point, we have used a heuristic in which specific bands are described as “rhythms” or “oscillations,” which implies the converse features “noise” and “arrhythmia” exist. While there has been immense utility in using this approach, there are distinct differences between biology and the imperfect analogy that leads to ambiguity or misunderstanding in the scientific interpretation of the results (Chapter 2 of Başar 2012). This has been briefly mentioned before by Wang and Buzsaki “…although the word ‘rhythm’ readily conjures up the picture of a clock, gamma rhythms occur in relatively short bursts and are quite variable in frequency…” (Buzsaki and Wang, 2012). The notion of a rhythm-to-clock relationship is most likely tied to the power-frequency representation in time-series decompositions. This was initially described as the “Fourier Fallacy” in which one assumes, based on the power spectra, that all the necessary frequencies in the power spectra occur as periodic sine waves in the brain (Jasper, 1948; n.b., this applies to any method which provides a “one power to one frequency” relationship). Indeed, the concept of a unique neurobiological generator ascribed to each Fourier frequency is more magic than reality (He, 2014). However, the gap between the abstract representation of a spectral decomposition and neurobiology has yet to be fully fleshed out. Therefore, the field reaches a crossroads where we are either left to (1) expand on the notion of Wang and Buzsaki (2012) and explore the idea that the interrelated frequencies in the LFP are primarily the consequence of synaptic transmembrane currents from a local network when receiving a barrage of input or (2) whole-heartedly buy into the idea that there are divisions to be made between neural rhythms and neural noise.

The power spectral density is a low-dimensional abstraction of the actual raw time series, collapsing the underlying time-series to a decimation of sine waves. Any such transformation requires significant consideration regarding the relationship of the raw data to the algorithmic lens (Rosen, 1991). One hypothesis suggests the peaks above the 1/f slope are true neurobiological oscillators, whereas the slope itself is the consequence of a distinct broadband, arrhythmic activity (He, 2014) or similarly, rhythmicity superimposed onto a wide-band, noisy background (Bullock et al., 2003). Along these lines, analytical toolboxes have been released that claim to dissect the periodic components from the aperiodic components of the LFP (Donoghue et al., 2020). However, this perspective falls to a different sort of Fourier Fallacy in which a dichotomous “periodic” vs. “aperiodic” division based on the idea that the underlying neurobiology has two opposing physiological patterns: one that makes a pure rhythm (peaks in the power spectra) and another that makes noise (the 1/f slope; Voytek et al., 2015). The major flaw in this perspective is that it over interprets the abstract representation of a power spectral density plot, assuming that it provides more information than what can be observed in the raw LFP trace. Specifically, any measure of “rhythmicity” in the nervous system should not only measure power, but the degree to which their phase assignment of each sine wave aligns. As an exercise, conduct a Fourier decomposition on a musical concerto, maintain the power across each sine wave but randomize their phases and listen to the time-series recovered using the inverse Fourier transform. The composition falls into discord. Any rhythmicity in the musical time-series is effectively destroyed while the power spectral density retains the same exact form. Therefore, it becomes evident that the power spectral density is an incomplete tool to make a rhythm vs. noise division in any time series. With respect to neurophysiology, the division misinterprets the abstract decomposition for a literal representation, obfuscating the biophysics of the system.

Wang and Buzsaki provided a glimpse into an alternative hypothesis in which the nervous system works through evolving unique spatio-temporal patterns (Buzsaki and Wang, 2012). These spatio-temporal patterns may sometimes manifest as easy-to-identify rhythms. However, the nervous system did not evolve to perfect “clockwork operations”. It should be appreciated that a single synaptic event can have rhythmic entrainment to both “theta” and “gamma” bands, challenging the heuristic of independence across rhythms. Instead, the dynamic patterns in the LFP are closely related to how the activity spatio-temporally evolves a reentrant network (Berg et al., 2019; Maurer and Nadel, 2021). While there is comfort in giving independence to specific patterns like theta and gamma, neurobiology makes no such distinction. To the neuroscientist, the Greek letters have meaning with respect to biophysical scale and the neurotransmitter shaping their interactions (among other features). The nervous system however is not self-aware of actions within a single frequency band. To relate a specific band to a behavior or cognitive function first and foremost requires a neurobiological description (what neurobiological or synaptic events lead to the increase in 80 Hz activity?) rather than a psychological, correlational answer (attention caused the increase in the 80 Hz band). The Greek letter-defined rhythms, while helpful in colloquial discussion, gave a platform to assign them individual tasks, such as synchronizing, entraining, buffering memory, and/or being a physiological readout of psychological function—a personification of sorts that speaks to the danger of taking the “rhythm” heuristic too far. Instead, it should be appreciated that the LFP is related to the non-linear spatio-temporal patterns that occur within a network of densely interconnected neurons, or more specifically, the LFP is primarily a biophysical product of the synaptic transmembrane currents associated with these dynamic patterns. In this reinterpretation, theta is the propagation of activity along a large “macroscale” loop. Nested within this large loop are many smaller loops in which the activity can be reciprocally coordinated on the mesoscale, supporting faster rhythms, such as gamma (Buzsaki and Draguhn, 2004; Buzsaki, 2006). The scales—and the names that define them- are not orthogonal but intricately coupled. The nervous system, however, does not care should a “peak” appear in the power spectra of the LFP.

Therefore, we offer to replace one heuristic (“rhythms,” “oscillators,” “noise,” “arrhythmia”) with another system defined by forcing and nested loops of multiple scales: the cardiovascular system. Should one measure the velocity of a red blood cell in a capillary, the time-series itself will exhibit the dominant frequency of the heartbeat (macroscale forcing event). As heartbeats are “slow charge, rapid discharge” event, the large amplitude changes in velocity will exhibit a significant deviation from a sinusoid. Furthermore, this forcing occurs as a cascade from macro to micro scale, from arteries through arterioles to capillaries. Thus, blood cell velocity will be subjected to other influences, such as friction as a result of running into the other blood cells or the vascular walls or even form high-frequency turbulent eddies (e.g., partial occlusion). This jostling can be identified in the velocity profile as being a repeatable, low amplitude-high-frequency event coupled to the macroscale heartbeat frequency. Finally, decomposing the red blood cell velocity would reveal a power spectra density eerily similar to the hippocampus, complete with a fundamental frequency, harmonics, and a 1/f background (see Figure 2B of Harlepp et al., 2017). In fact, one may suspect that the influence of pentobarbital on the power spectra of red blood cell velocity would parallel the degradation observed in the LFP, with the highest frequencies succumbing first. Therefore, while Fourier decomposition is certainly a useful tool in time-series analysis, defining the presence of an oscillation as a peak above the background and the absence as having no peak along the 1/f background as “aperiodic” from the power spectra has a peculiar and contracted relevance. The cardiovascular system never evolved to make rhythms or arrhythmic activity but takes advantage of a turbulent energy cascade across scales to move blood cells (Saqr et al., 2020).

As “…the EEG reflects the ‘average’ behavior of neurons” (Buzsaki, 2006, p. 129), the analogy above has a direct relation to neurophysiological theories incorporating the classical physics theory of turbulence into the description of the LFP (Sheremet et al., 2019b; Deco and Kringelbach, 2020). An action potential of a single neuron represents the smallest spatio-temporal event, the microscale component, that resides in nested loops of multiple scales. The LFP is the aggregate activity related to activity moving through multiple loops of different scales simultaneously. The movement of the activity through the nervous system is, from this perspective, a unitary process where activity “chases is own tail” through reentrant loops (Hebb, 1958). Cross-frequency dependence becomes the rule rather than the exception. Certainly, there may be activity that expresses as a peak above the 1/f background, such as theta, which comprises the movement of activity on the macroscale. However, oscillations like gamma rarely peak above the background (Zhou et al., 2019) and yet also describe the mesoscale volleys of activity governed by interneurons. Rather than making false dichotomies between “non-rhythm/rhythm,”“present/absent,”“periodic/aperiodic” a new appreciation is required in which the LFP is a reflection of the underlying evolution of spatio-temporal patterns within a densely interconnected network of neurons.

## Data Availability Statement

The raw data supporting the conclusions of this article are available for download without reservation here: https://datadryad.org/stash/dataset/doi:10.5061/dryad.vmcvdncs6.

## Ethics Statement

The animal study was reviewed and approved by University of Florida Institute of Animal Care and Use Committee.

## Author Contributions

AS and AM came up with the idea of investigating the spectrum evolution with decreasing energy and participated in the entire process of this study. JK and ND performed the surgery, conducted experiments, and collected the data. YZ conducted the data analysis and made the figures. CM and SB provided insights into the interpretation of the results. All authors made contributions to writing the manuscript.

## Conflict of Interest

The authors declare that the research was conducted in the absence of any commercial or financial relationships that could be construed as a potential conflict of interest.

## Supplementary Material

Supplementary Figure 1 Background spectrum of hippocampal LFP. The background spectrum is indicated as the gray dashed line. Theta, theta harmonics, gamma, and ripple will develop on the background spectrum at different behaving states.

Supplementary Figure 2 Hippocampal lamination of five rats with current source density (CSD) analysis. The CSD analyses were triggered on the maximum positive-going ripple in the pyramidal cell layer. DOF, Degree of freedom, indicating the number of ripple events. Strong sharp wave sources and sinks can be observed at pyramidal layer (Pyr), stratum radiatum (Rad), lacunosum-moleculare (LM), and upper granule layer (G). Embedded high-frequency ripples can be spotted at strata Pyr and Rad.

Supplementary Figure 3 Coherent high-frequency bursts (>128 Hz) during stage 1. **(A)** Power evolution of frequency components over 128 Hz during early stage of euthanasia. Four instances with strong (2nd and 3rd) or weak (1st and 4th) high-frequency power were marked with dashed lines. **(B)** Raw LFPs of four layers (Pyr, Rad, LM, and MEC) at instances marked by dashed lines in **(A)**. **(C)** LFPs band-pass filtered in frequency range 140–160 Hz at instances marked by dashed lines in **(A)**. At instances with strong high-frequency power (2nd and 3rd columns), synchronized wave envelopes can be observed across layers. **(D)** Evolution of coherence between Pyr and LM. **(E)** Evolution of coherence between Pyr and MEC layer. **(F)** Evolution of phase lag between Pyr and LM. **(G)** Evolution of phase lag between Pyr and MEC layer. According to **(D–G)**, during the stage 1 of degradation, the cross-spectrum at high-frequency range (>128 Hz) is dominated by zero phase-lag high-coherent events.

Supplementary Figure 4 Similar with Figure 2. Data from rat 730.

Supplementary Figure 5 Similar with Figure 2. Spectrum degradation in hippocampus. Data from rat 829.

Supplementary Figure 6 Similar with Figure 2. Spectrum degradation in hippocampus. Data from rat 889.

Supplementary Figure 7 Similar with Figure 2. Spectrum degradation in hippocampus. Data from rat 1074.

Supplementary Figure 8 Similar with Figure 3. Data from rat 730.

Supplementary Figure 9 Similar with Figure 3 without cross region coherence and bicoherence. Data from rat 829.

Supplementary Figure 10 Similar with Figure 3 without cross region coherence and bicoherence. Data from rat 889.

Supplementary Figure 11 Similar with Figure 3 without cross region coherence and bicoherence. Data from rat 1074.
