# Supplementary figures and images for: Spectrum Degradation of Hippocampal LFP During Euthanasia
Source: Front Syst Neurosci. 2021 Apr 23;15:647011. doi: 10.3389/fnsys.2021.647011 (PMC8102791; doi:10.3389/fnsys.2021.647011)

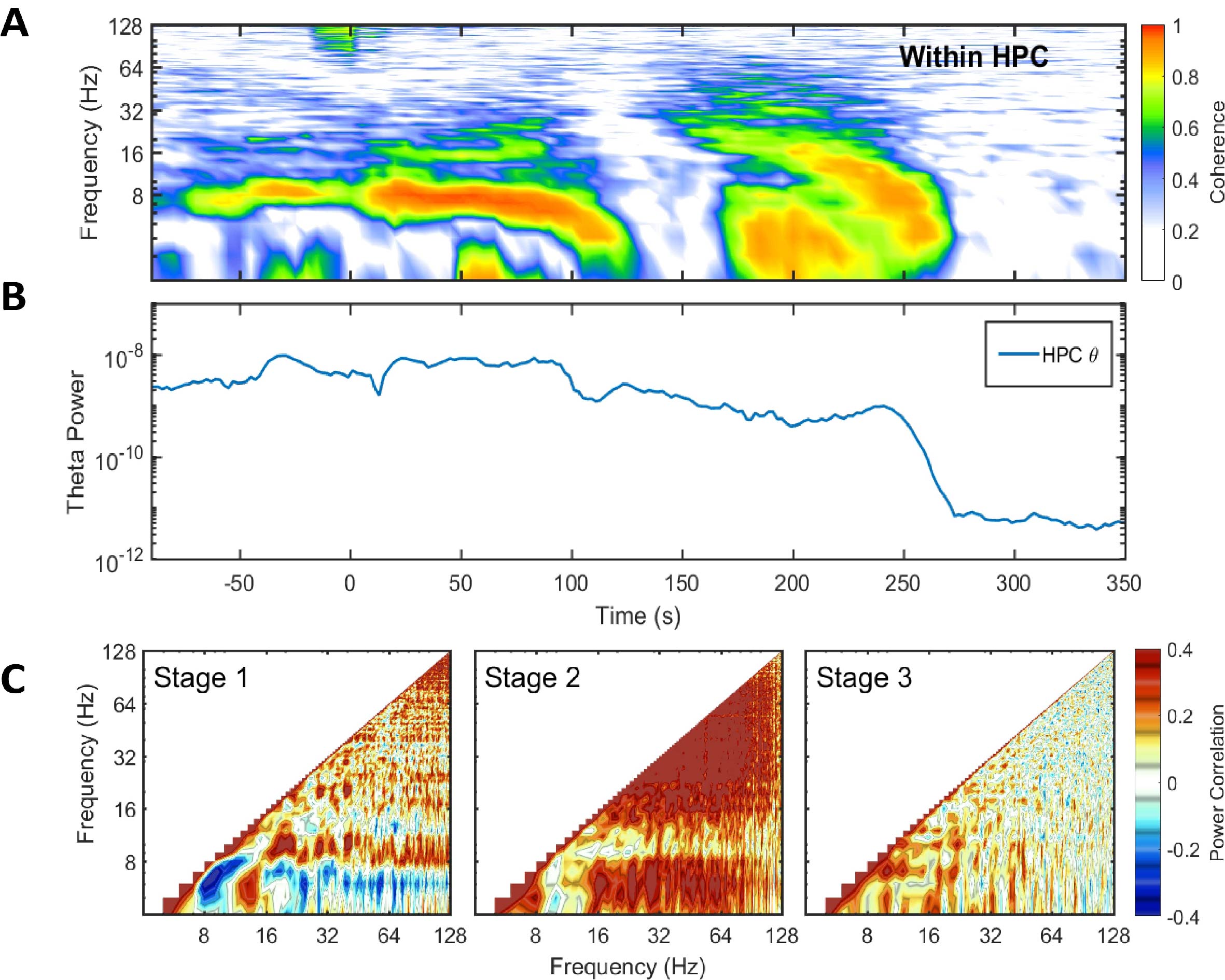

Supplement: Supplementary Figure 1 — Background spectrum of hippocampal LFP. The background spectrum is indicated as the gray dashed line. Theta, theta harmonics, gamma, and ripple will develop on the background spectrum at different behaving states. [file Image_1.JPEG]

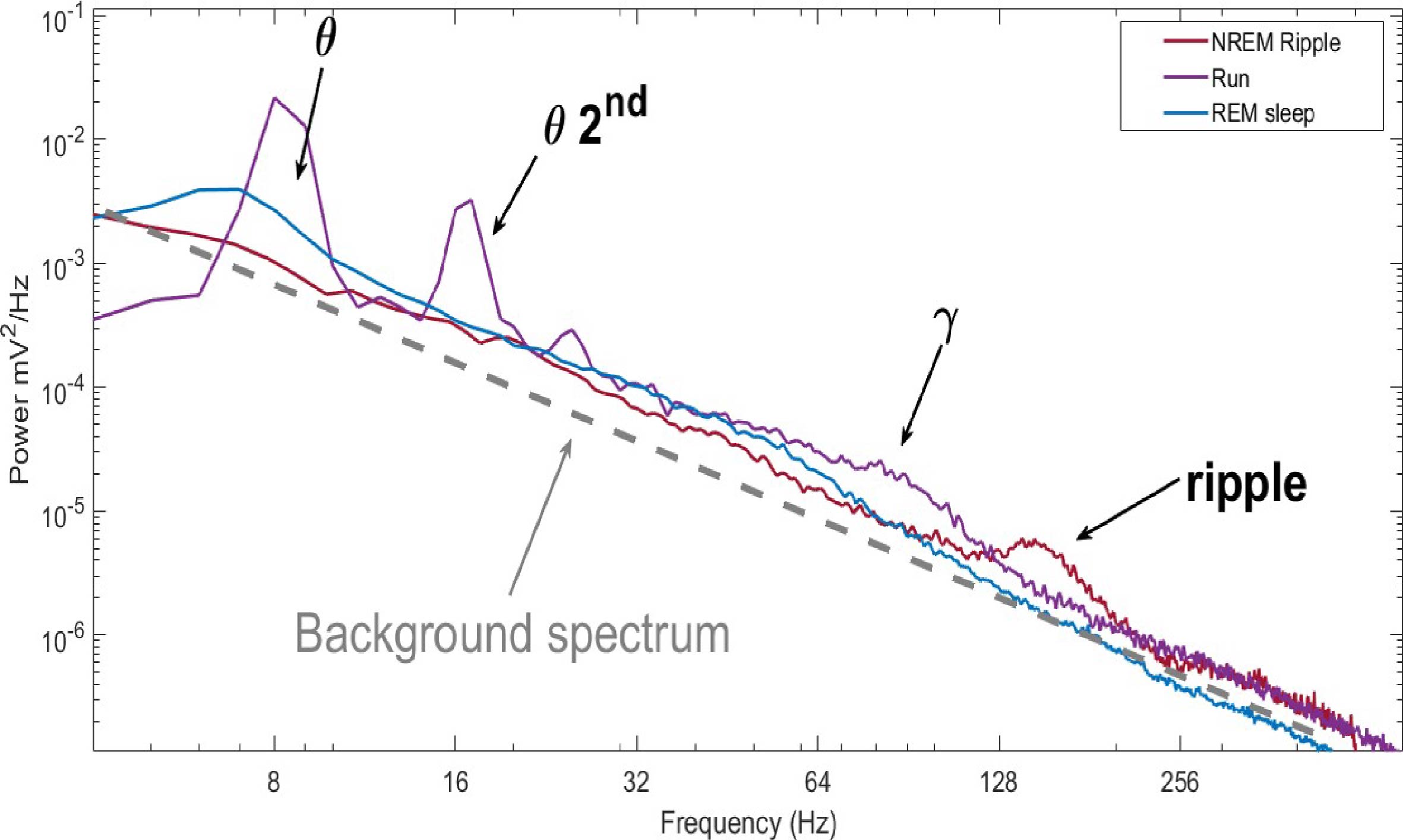

Supplement: Supplementary Figure 2 — Hippocampal lamination of five rats with current source density (CSD) analysis. The CSD analyses were triggered on the maximum positive-going ripple in the pyramidal cell layer. DOF, Degree of freedom, indicating the number of ripple events. Strong sharp wave sources and sinks can be observed at pyramidal layer (Pyr), stratum radiatum (Rad), lacunosum-moleculare (LM), and upper granule layer (G). Embedded high-frequency ripples can be spotted at strata Pyr and Rad. [file Image_2.JPEG]

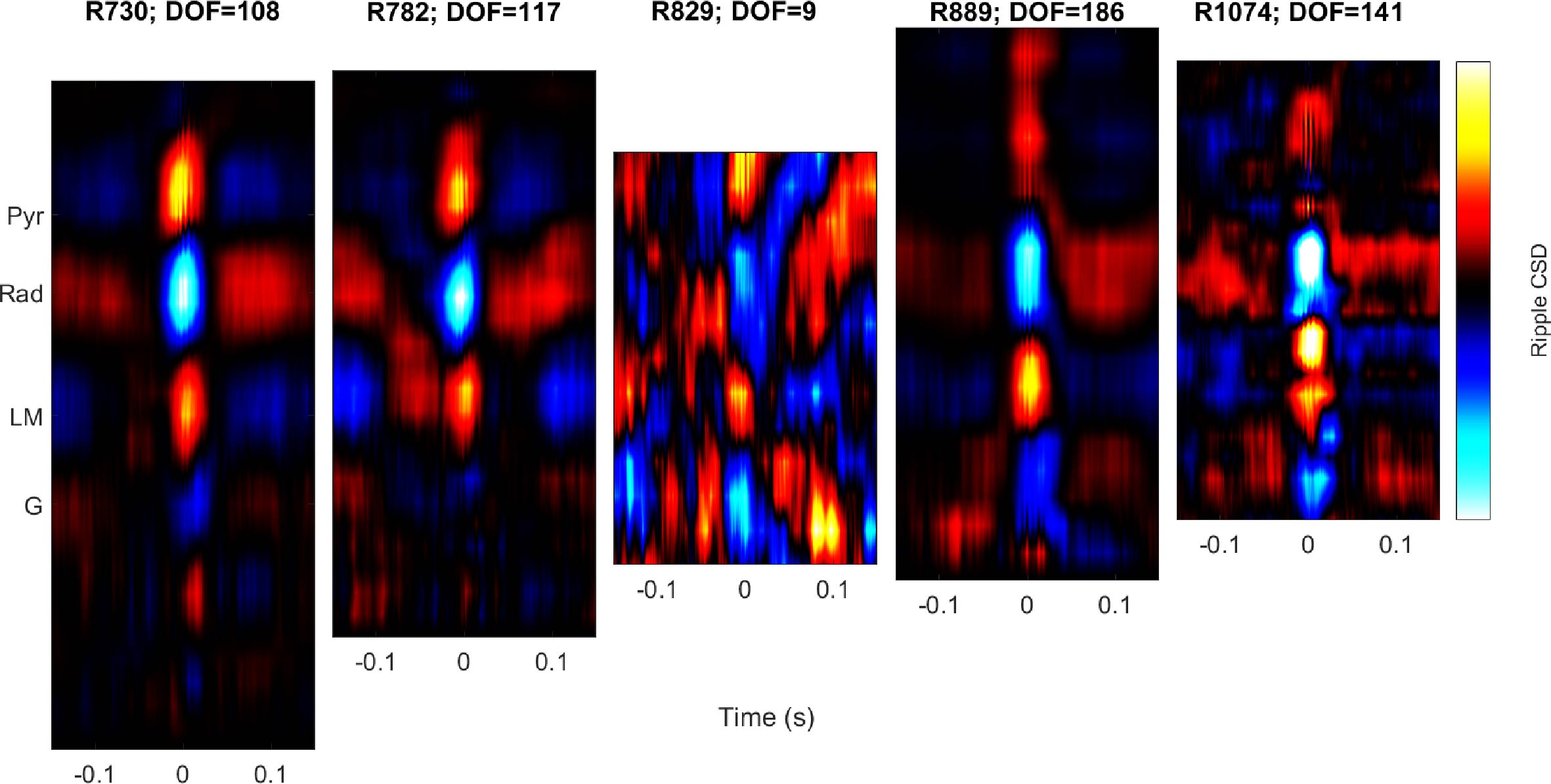

Supplement: Supplementary Figure 3 — Coherent high-frequency bursts (>128 Hz) during stage 1. (A) Power evolution of frequency components over 128 Hz during early stage of euthanasia. Four instances with strong (2nd and 3rd) or weak (1st and 4th) high-frequency power were marked with dashed lines. (B) Raw LFPs of four layers (Pyr, Rad, LM, and MEC) at instances marked by dashed lines in (A). (C) LFPs band-pass filtered in frequency range 140–160 Hz at instances marked by dashed lines in (A). At instances with strong high-frequency power (2nd and 3rd columns), synchronized wave envelopes can be observed across layers. (D) Evolution of coherence between Pyr and LM. (E) Evolution of coherence between Pyr and MEC layer. (F) Evolution of phase lag between Pyr and LM. (G) Evolution of phase lag between Pyr and MEC layer. According to (D–G), during the stage 1 of degradation, the cross-spectrum at high-frequency range (>128 Hz) is dominated by zero phase-lag high-coherent events. [file Image_3.JPEG]

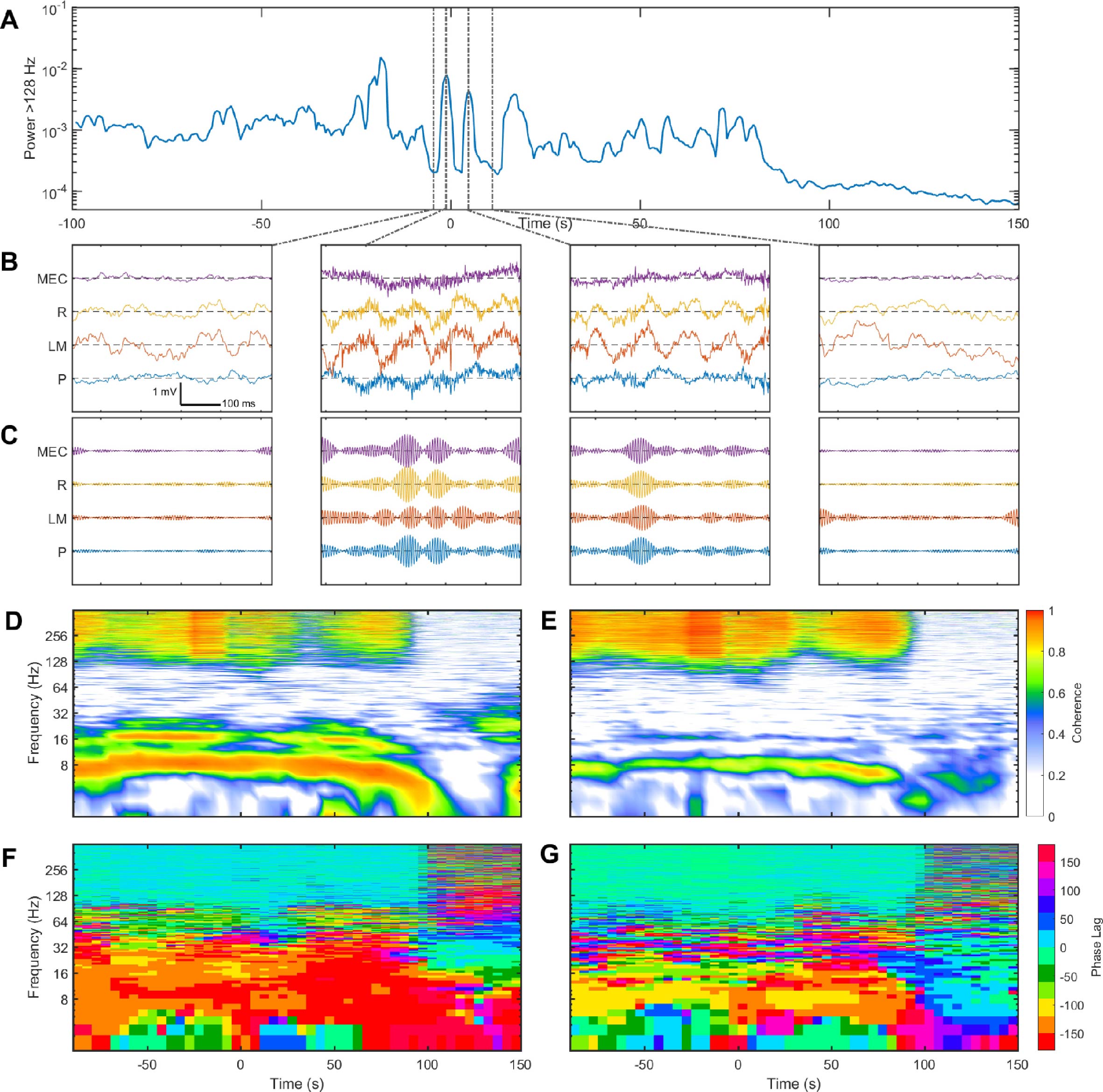

Supplement: Supplementary Figure 4 — Similar with Figure 2. Data from rat 730. [file Image_4.JPEG]

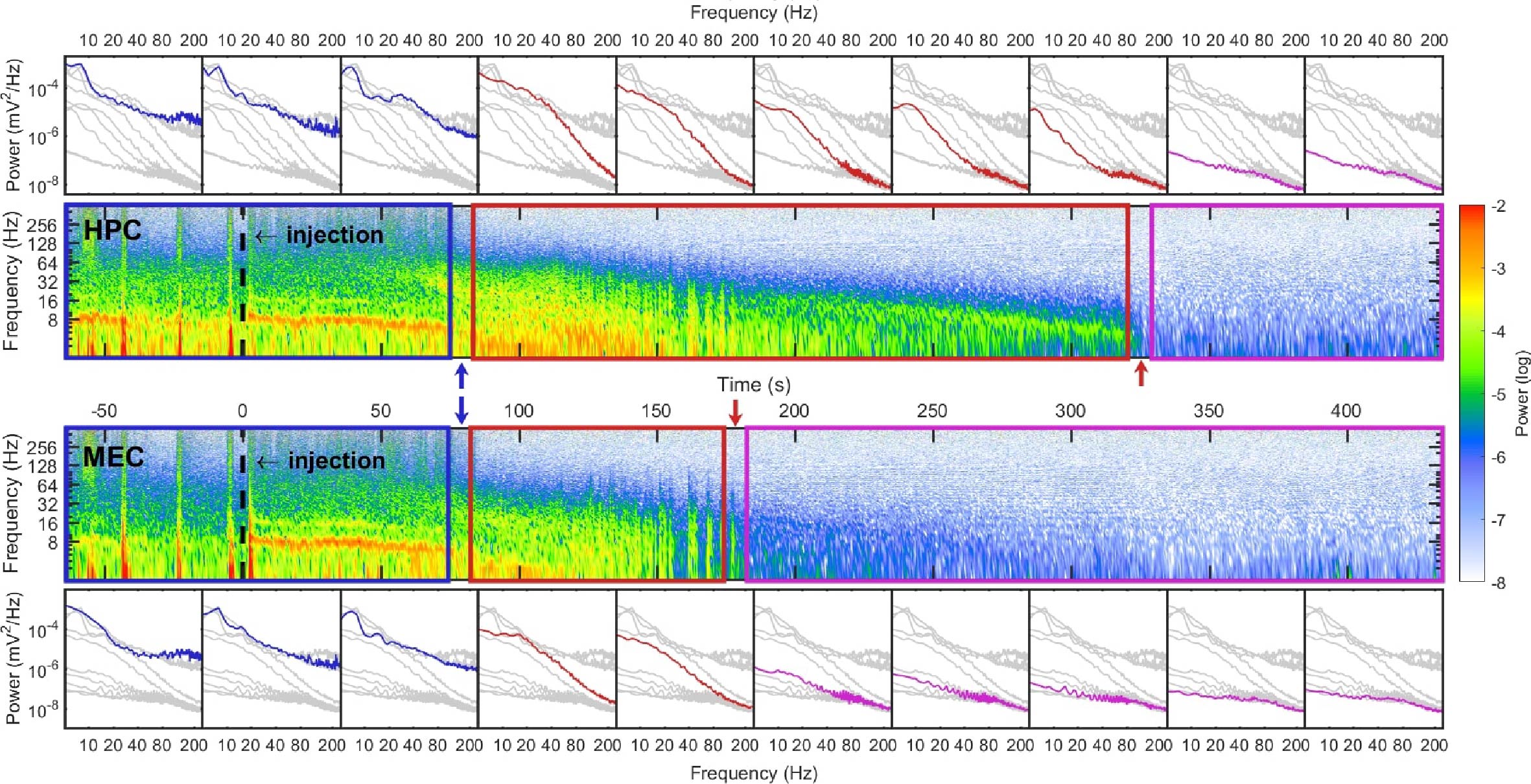

Supplement: Supplementary Figure 5 — Similar with Figure 2. Spectrum degradation in hippocampus. Data from rat 829. [file Image_5.JPEG]

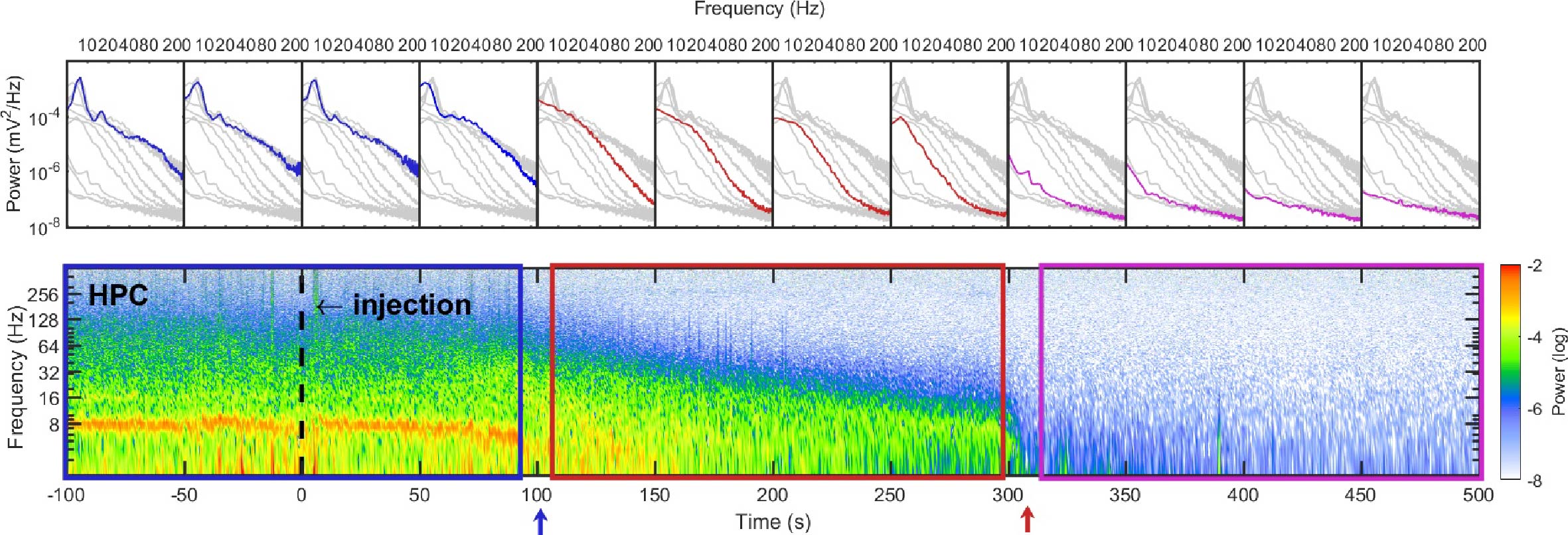

Supplement: Supplementary Figure 6 — Similar with Figure 2. Spectrum degradation in hippocampus. Data from rat 889. [file Image_6.JPEG]

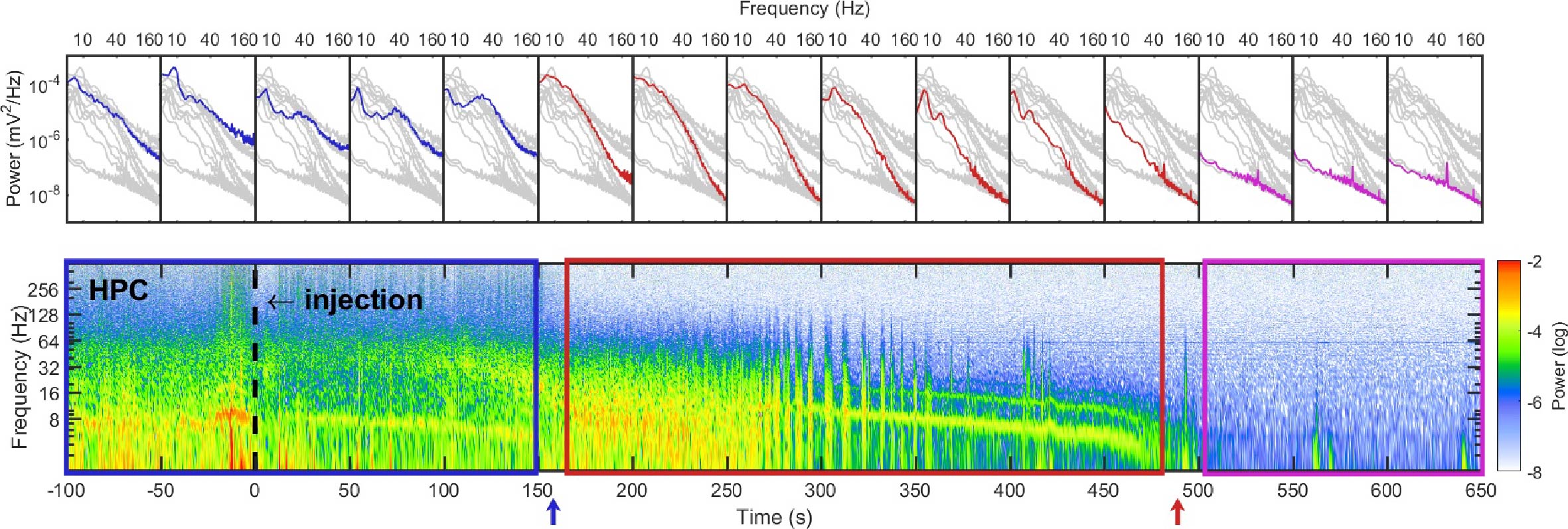

Supplement: Supplementary Figure 7 — Similar with Figure 2. Spectrum degradation in hippocampus. Data from rat 1074. [file Image_7.JPEG]

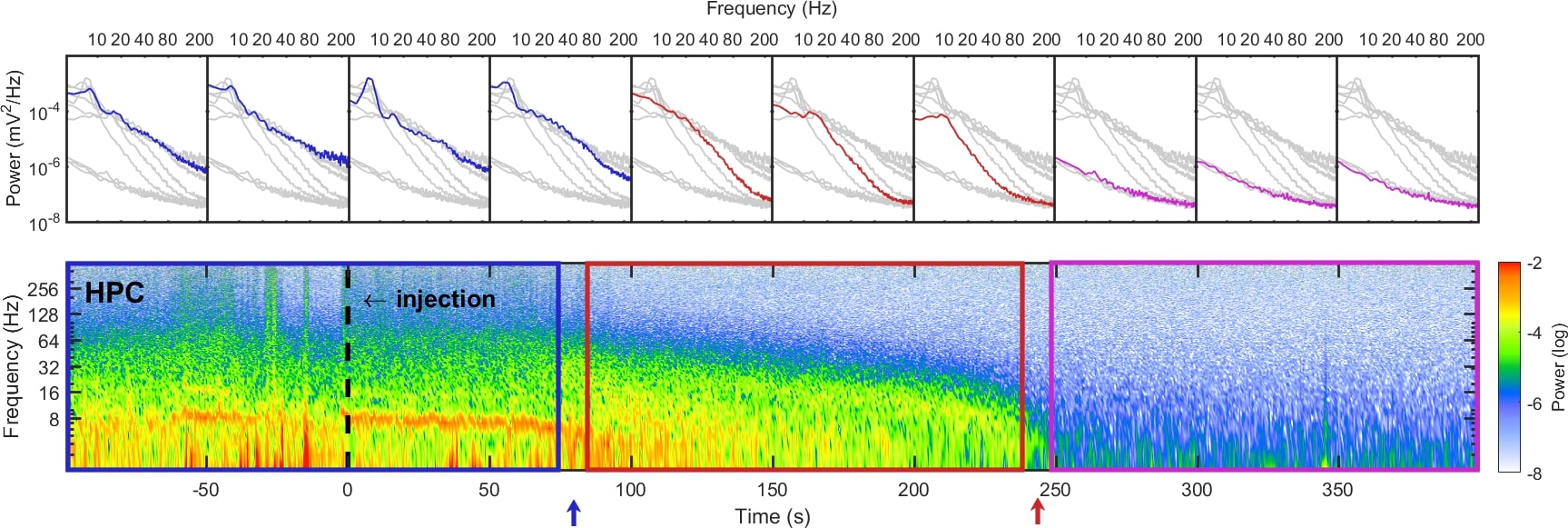

Supplement: Supplementary Figure 8 — Similar with Figure 3. Data from rat 730. [file Image_8.JPEG]

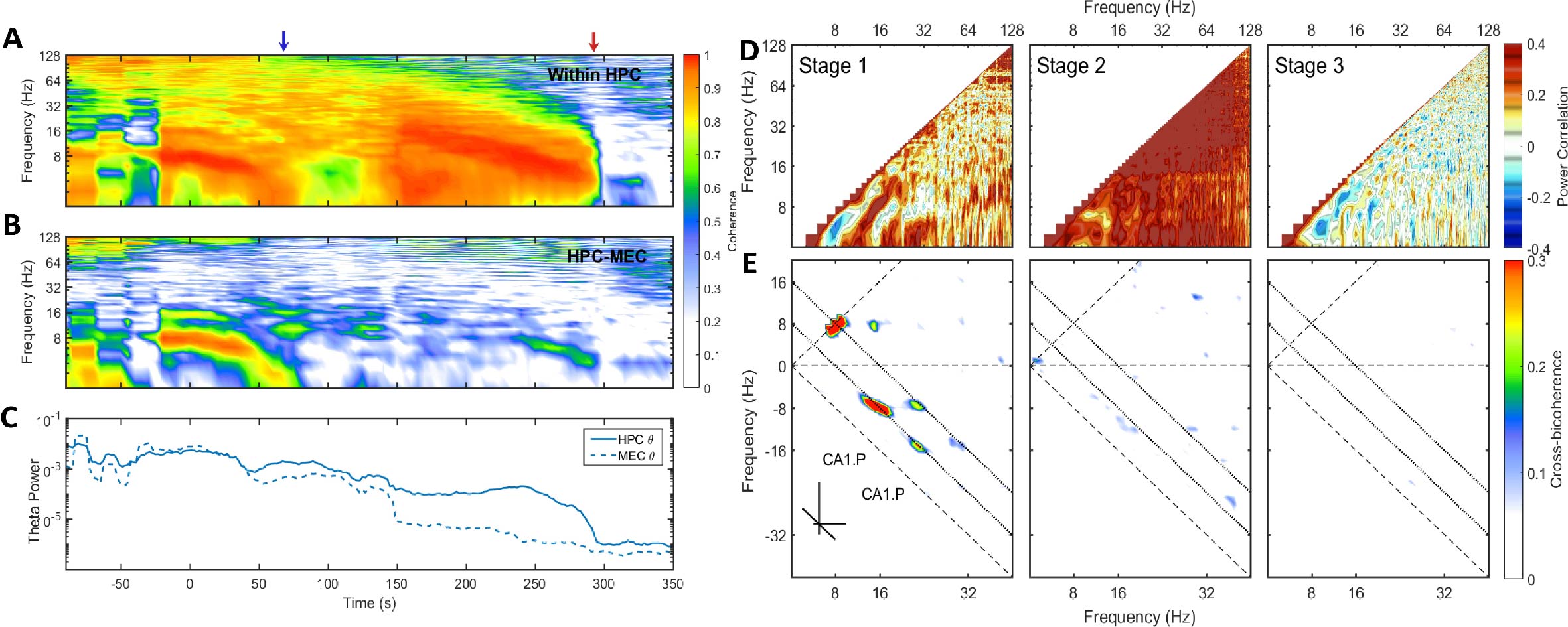

Supplement: Supplementary Figure 9 — Similar with Figure 3 without cross region coherence and bicoherence. Data from rat 829. [file Image_9.JPEG]

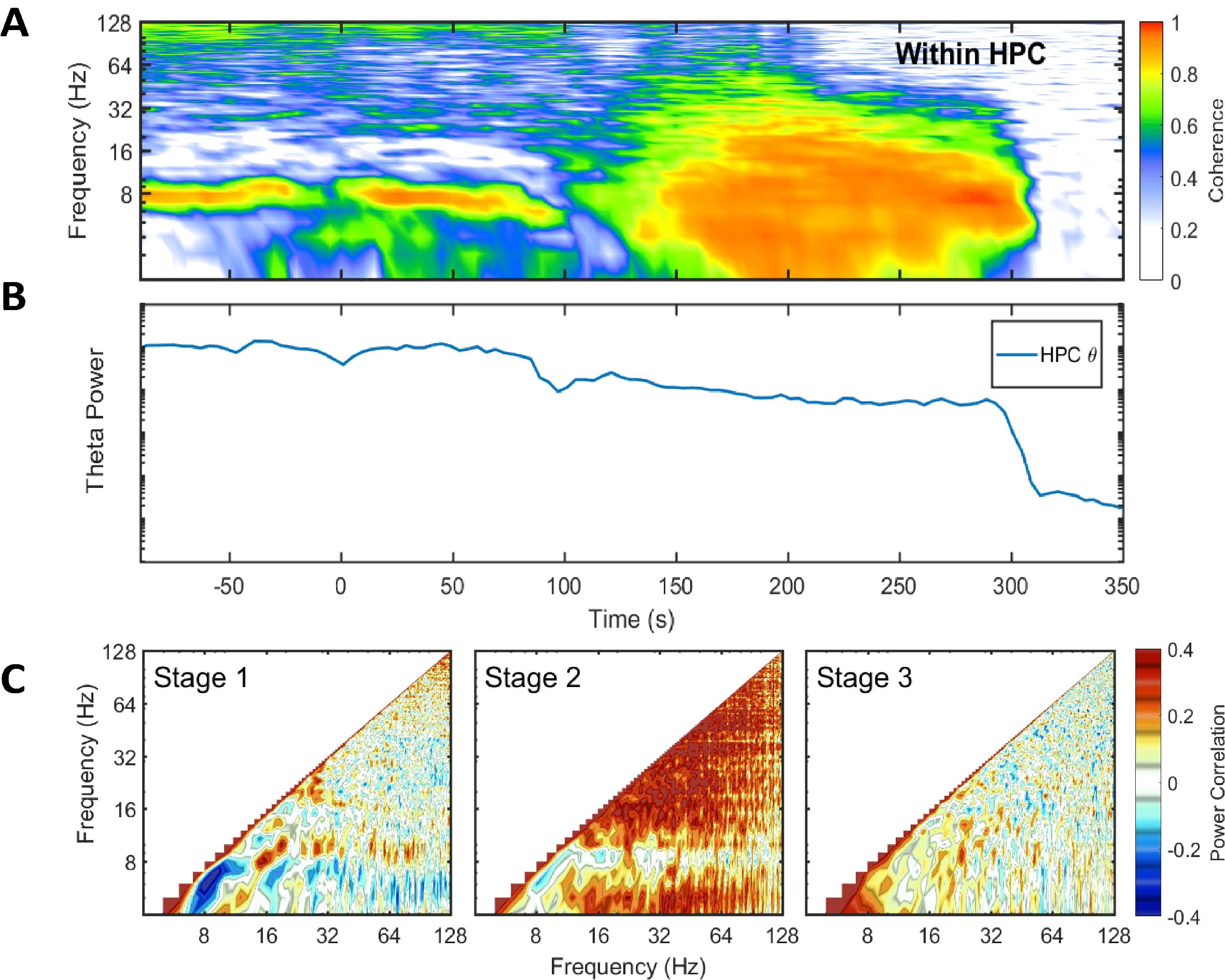

Supplement: Supplementary Figure 10 — Similar with Figure 3 without cross region coherence and bicoherence. Data from rat 889. [file Image_10.JPEG]

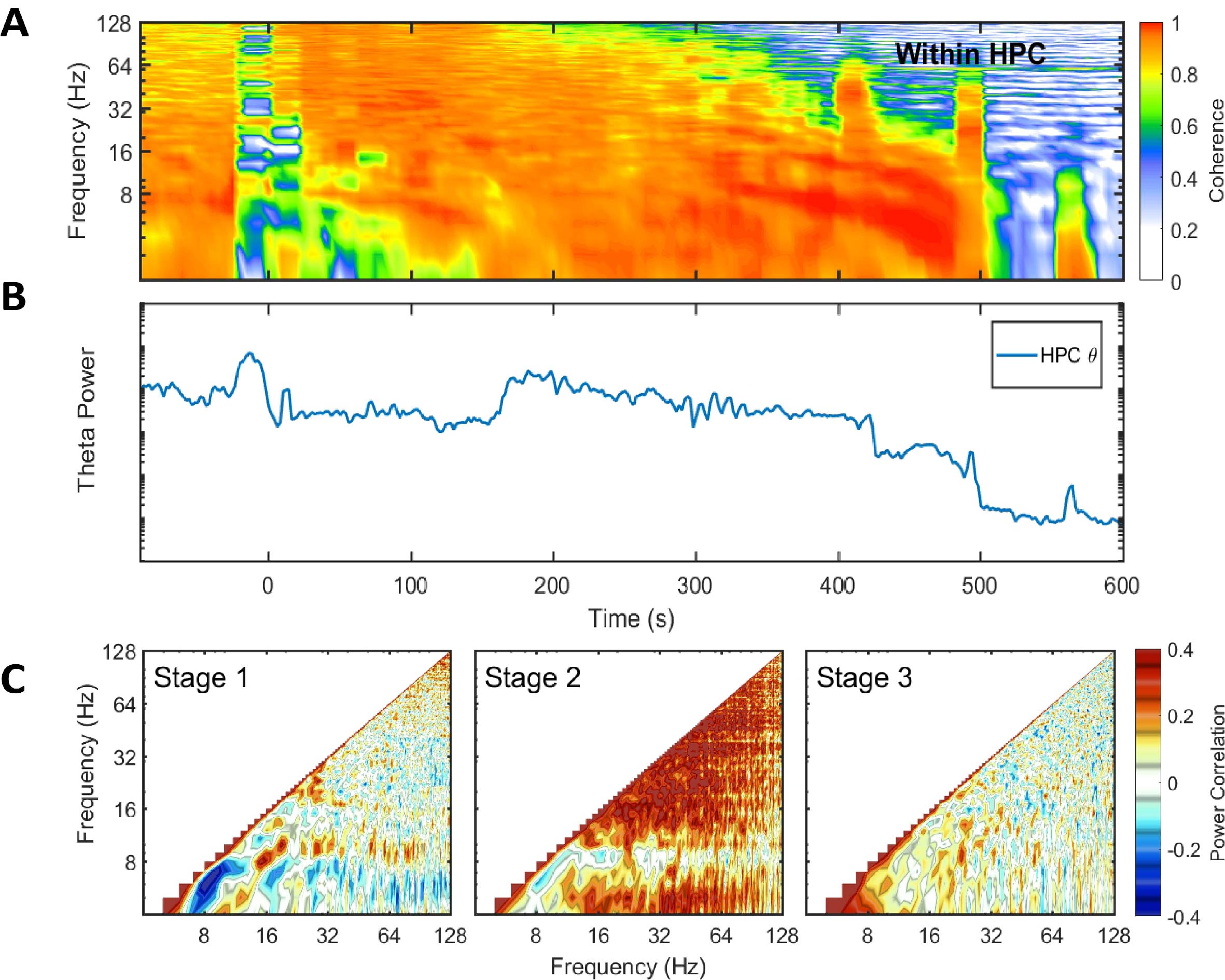

Supplement: Supplementary Figure 11 — Similar with Figure 3 without cross region coherence and bicoherence. Data from rat 1074. [file Image_11.JPEG]
